# Pro-Oxidant Auranofin and Glutathione-Depleting Combination Unveils Synergistic Lethality in Glioblastoma Cells with Aberrant Epidermal Growth Factor Receptor Expression

**DOI:** 10.3390/cancers16132319

**Published:** 2024-06-25

**Authors:** Elvis Martinez-Jaramillo, Fatemeh Jamali, Farah H. Abdalbari, Bassam Abdulkarim, Bertrand J. Jean-Claude, Carlos M. Telleria, Siham Sabri

**Affiliations:** 1 Experimental Pathology Unit, McGill University, Montréal, QC H3A 2B4, Canada; elvis.martinez-jaramillo@mail.mcgill.ca (E.M.-J.); farah.abdalbari@mail.mcgill.ca (F.H.A.); 2 Cancer Research Program, Research Institute of the McGill University Health Centre, Montréal, QC H4A 3J1, Canada; fatemeh.jamali@mail.mcgill.ca (F.J.); bassam.abdulkarim@mcgill.ca (B.A.); 3 Department of Oncology, McGill University Health Centre, Montréal, QC H4A 3J1, Canada; 4 Department of Medicine, Faculty of Medicine and Health Sciences, McGill University, Montréal, QC H4A 3J1, Canada; bertrandj.jean-claude@mcgill.ca; 5 Cancer Drug Research Laboratory, Metabolic Disorders and Complications Program, Centre for Translational Biology, Research Institute of the McGill University Health Centre, Montréal, QC H4A 3J1, Canada

**Keywords:** glioblastoma, auranofin, thioredoxin reductase (TrxR1), EGFR, EGFRvIII, reactive oxygen species (ROS), oxidative stress, glutathione (GSH), antioxidant, drug repurposing

## Abstract

**Simple Summary:**

Glioblastoma (GBM) is a fatal brain cancer. Over 50% of GBM tumors overexpress EGFR, a protein involved in tumor progression and drug resistance. The thioredoxin and glutathione antioxidant systems counteract oxidative stress, leading to drug resistance in GBM. In this study, we aimed to investigate the impact of EGFR overexpression on the response to the thioredoxin inhibitor and arthritis medication, auranofin and glutathione inhibitor L-buthionine-sulfoximine (L-BSO). Using EGFR-negative and two derived EGFR-overexpressing GBM cells, we validated the hypothesis according to which the auranofin and L-BSO combination reach the lethal threshold of oxidative stress. Auranofin increased oxidative stress, DNA damage, and cell killing while decreasing EGFR. Auranofin and L-BSO combination induced further cell killing, revealing the vulnerability of EGFR in response to co-targeting the thioredoxin and glutathione systems. Our findings unravel the significance of impairing antioxidant defense in EGFR-driven cancer and repurposing auranofin and L-BSO treatment to improve the dismal survival of patients with GBM.

**Abstract:**

Glioblastoma (GBM) is the most prevalent and advanced malignant primary brain tumor in adults. GBM frequently harbors epidermal growth factor receptor (EGFR) wild-type (*EGFRwt*) gene amplification and/or *EGFRvIII* activating mutation. EGFR-driven GBM relies on the thioredoxin (Trx) and/or glutathione (GSH) antioxidant systems to withstand the excessive production of reactive oxygen species (ROS). The impact of EGFRwt or EGFRvIII overexpression on the response to a Trx/GSH co-targeting strategy is unknown. In this study, we investigated Trx/GSH co-targeting in the context of EGFR overexpression in GBM. Auranofin is a thioredoxin reductase (TrxR) inhibitor, FDA-approved for rheumatoid arthritis. L-buthionine-sulfoximine (L-BSO) inhibits GSH synthesis by targeting the glutamate–cysteine ligase catalytic (GCLC) enzyme subunit. We analyzed the mechanisms of cytotoxicity of auranofin and the interaction between auranofin and L-BSO in U87MG, U87/EGFRwt, and U87/EGFRvIII GBM isogenic GBM cell lines. ROS-dependent effects were assessed using the antioxidant N-acetylsteine. We show that auranofin decreased TrxR1 activity and increased ROS. Auranofin decreased cell vitality and colony formation and increased protein polyubiquitination through ROS-dependent mechanisms, suggesting the role of ROS in auranofin-induced cytotoxicity in the three cell lines. ROS-dependent PARP-1 cleavage was associated with EGFRvIII downregulation in U87/EGFRvIII cells. Remarkably, the auranofin and L-BSO combination induced the significant depletion of intracellular GSH and synergistic cytotoxicity regardless of EGFR overexpression. Nevertheless, molecular mechanisms associated with cytotoxicity were modulated to a different extent among the three cell lines. U87/EGFRvIII exhibited the most prominent ROS increase, P-AKT(Ser-473), and AKT decrease along with drastic EGFRvIII downregulation. U87/EGFRwt and U87/EGFRvIII displayed lower basal intracellular GSH levels and synergistic ROS-dependent DNA damage compared to U87MG cells. Our study provides evidence for ROS-dependent synergistic cytotoxicity of auranofin and L-BSO combination in GBM in vitro. Unraveling the sensitivity of EGFR-overexpressing cells to auranofin alone, and synergistic auranofin and L-BSO combination, supports the rationale to repurpose this promising pro-oxidant treatment strategy in GBM.

## 1. Introduction

Glioblastoma, previously known as Glioblastoma multiforme (GBM), designates isocitrate dehydrogenase (*IDH*)-wildtype diffuse glioma in adults [1]. It is the most common and advanced primary malignant brain tumor, which infers a very poor prognosis for an incurable cancer [2,3]. For almost two decades, surgical resection, radiotherapy, and temozolomide (TMZ) chemotherapy remained the mainstay GBM treatment, with approximately 15 months for median GBM patient survival [4].

Epidermal growth factor receptor (*EGFR*) gene alterations, including amplification, mutation, rearrangement, and altered splicing, have been reported in 57% of GBMs [5]. *EGFR* amplification is among the few molecular alterations currently used to confirm GBM *IDH*-wildtype grade 4 diagnosis [1]. EGFR, the most commonly amplified receptor tyrosine kinase in GBM [6], can be overexpressed and/or mutated in GBM [7]. One of the most frequent mutations of the receptor is the EGFR variant III (EGFRvIII), a truncated version constitutively active in the absence of ligand [8]. EGFRvIII expression has been associated with aggressive tumor progression [9]. *EGFR* alterations with subsequent protein overexpression activate a plethora of signaling pathways, including PI3K/Akt and extracellular signal-regulated kinase (ERK1/2), which promote tumor proliferation, resistance to apoptosis, chemoradioresistance, and GBM tumor recurrence [10]. Accordingly, the therapeutic significance of EGFR as a clinical target instigated strategies to counteract the oncogenic effects of *EGFR* alterations, such as small-molecule tyrosine kinase inhibitors, antibodies, and vaccines. However, thus far, clinical trials have not shown a significant improvement in the overall survival of patients with GBM [11,12].

Increasing oxidative stress to reach the threshold of irreversible lethal oxidative damage has been used as a potential strategy for cancer treatment [13,14,15]. Reactive oxygen species (ROS) designate highly reactive oxygen-containing molecules generated during aerobic cellular mitochondrial respiration and metabolic reactions that produce cellular energy. Increased ROS levels in cancer cells results from the high proliferative rate and metabolic activity of cancer cells compared to normal non-transformed cells. This differential ROS level enables the use of pro-oxidant strategies to selectively increase ROS to a cytotoxic threshold level for cancer cells without affecting normal cells [16].

The ROS “threshold concept” implies the ability of cancer cells to keep their intrinsic ROS levels at a threshold favorable for tumor growth below the threshold of excessive cytotoxic ROS levels [17]. To this end, cancer cells rely on increasing the expression and/or activity of the main endogenous antioxidants, glutathione (GSH), and the thioredoxin (Trx) systems, as key adaptive ROS-scavenging mechanisms via a mechanism called redox resetting [18,19]. The Trx system comprises the selenoenzyme thioredoxin reductase (TrxR), its substrate Trx, and NAPDH, which serves as a source of electrons to maintain Trx in a reduced state, essential for its ROS-scavenging function [20]. TrxR1 overexpression has been associated with poor prognosis and chemoresistance in many cancers, including GBM, thereby providing the rationale to target TrxR1 using electrophilic TrxR1 inhibitors [21]. Auranofin, a gold-based compound originally FDA-approved for rheumatoid arthritis, inhibits TrxR by directly interacting with the selenocysteine residue in the active redox center of TrxR. Studies targeting TrxR1 using auranofin have shown promising potent antitumor effects in different types of cancers [22]. Nonetheless, to overcome ROS increase following TrxR-targeted inhibition and to keep ROS below the lethal threshold, cancer cells activate “redox resetting” compensatory mechanisms to further increase the expression and/or antioxidant activity of the GSH system [18]. This includes the GSH, a non-enzymatic antioxidant tripeptide, and GSH-metabolizing enzymes, such as the glutamate–cysteine ligase catalytic (GCLC) subunit required for ligation of L-glutamate and L-cysteine in the first step of GSH biosynthesis [19]. Hence, co-targeting Trx/GSH systems should be implemented to reach the lethal ROS threshold in cancer cells.

Previous studies reported the role of ROS in EGFR activation and the correlation between ROS and EGFR in tumor progression and drug resistance [23,24,25,26,27]. Exposure to mild ROS levels induced an aberrant phosphorylation pattern and impaired EGFR trafficking and degradation, leading to ROS-mediated tumor progression [28]. Aberrant EGFRvIII expression in GBM and *EGFR* amplification in GBM stem cells have been associated with increased basal levels of ROS [29,30]. The crosstalk between EGFR and the GSH system directly inhibits apoptosis and increases resistance to electrophilic drugs in EGFR-positive GBM [31,32,33]. Hence, increased intrinsic basal ROS levels in EGFR-positive GBM and the crosstalk between EGFR and the GSH system underlie the rationale to investigate a pro-oxidant Trx/GSH co-targeting strategy in EGFR-positive GBM.

The impact of EGFR wild-type (EGFRwt) or EGFRvIII overexpression on the response to a pro-oxidant Trx/GSH co-targeting strategy to reach the lethal ROS threshold is currently unknown in GBM. In this work, we tested the hypothesis that exploiting the intrinsically high oxidative status associated with *EGFR* alterations may sensitize GBM EGFR-positive cells to a pro-oxidant strategy co-targeting the Trx/GSH antioxidant systems. Using GBM cell lines isogenic for EGFRwt and EGFRvIII, we provide the first evidence of auranofin-induced cytotoxicity through a ROS-dependent mechanism in EGFR-positive cells. We further demonstrate that auranofin, in combination with L-buthionine-sulfoximine (L-BSO), known to deplete GSH through inhibition of GCLC, has synergistic lethality in GBM cells irrespective of EGFRwt and EGFRvIII overexpression in vitro. Nonetheless, the molecular mechanisms associated with cytotoxic effects were modulated to a different extent between the three cell lines. These findings revealed a key role for GSH as a potential vulnerability encompassing EGFRwt and EGFRvIII for synergistic Trx/GSH co-targeting in GBM cell lines.

## 2. Materials and Methods

### 2.1. Cell Culture and Reagents

U87MG and its isogenic counterparts stably transfected to overexpress EGFR (U87/EGFRwt) or EGFRvIII (U87/EGFRvIII) GBM cell lines were generated using retroviral transfer of mutant receptor carrying neomycin resistance gene into U87MG cells [34,35,36,37]. Cells were maintained either in 1X DMEM (Cat. No. 11885092, Gibco, Life Technologies Corporation, Carlsbad, CA, USA) low glucose, l-glutamine, sodium pyruvate, phenol red; or 1X DMEM, (Cat No 319-010-CL, Wisent Inc., St-Jean-Baptiste, QC, Canada) supplemented with 10% fetal bovine serum (FBS) Premium US origin (Cat. No. 080-150, Wisent Inc.) 1X Penicillin-Streptomycin 50 U/mL (Cat. No. 15070063, Gibco) and incubated in 5% CO_2_ atmosphere at 37 °C. Cells were treated with dimethyl sulfoxide (DMSO; Cat. No BPBP231, Fisher Scientific Company, Fair Lawn, NJ, USA) as a vehicle control, auranofin (Cat. No 15316-25, Cayman Chemical Co., Ann Arbor, MI, USA), N-Acetyl cysteine (NAC; Cat. No A9165, Sigma, Saint Louis, MO, USA), or L-buthionine sulfoximide (L-BSO; Cat. No B2515, Sigma).

### 2.2. Cell Vitality

Cells growing at 70% confluency were harvested and seeded in triplicate in 96-well plates at a density of 2.5 × 10^3^ cells/well in supplemented DMEM medium and allowed to adhere overnight at 37 °C in 5% CO_2_. The cells were then treated with auranofin alone, NAC, or L-BSO at varying concentrations for 72 h. Cell vitality (a measure of cellular well-being as we reported earlier [38]) was assessed by adding 10 μL/well of 5 mg/mL MTT [3-(4,5-dimethylthiazol-2-yl)-2,5-diphenyltetrazolium bromide (Cat. No M6494, Invitrogen, Life Technologies Corporation, Carlsbad, CA, USA) in 1X Phosphate Buffered Saline (PBS; Cat. No 311-012, Wisent Inc.) solution. Cells were incubated for 4 h at 37 °C in 5% CO_2,_ where tetrazolium dye was reduced to insoluble formazan, and then 100 μL/well of 10% sodium dodecyl sulfate (SDS)/0.01 M HCl was added to stop the assay [39]. The absorbance was recorded at 570 nm on a microplate reader Bio-Tek Cytation 3 Multi-Mode Reader (Serial No. 131106B, Agilent, Santa Clara, CA, USA) following overnight incubation. Blank controls were subtracted, and the percentage of cell vitality was calculated relative to the control.

### 2.3. Total Count and Viability Assay

To determine the count and viability of cellular samples, triplicate cultures were trypsinized, separated by centrifugation at 1000× *g* for 3 min, and washed with 1X PBS. The cells were resuspended in the ViaCount reagent (Cat. No. MCH600103, EMD Millipore, Hayward, CA, USA), incubated at room temperature for 5 min, and analyzed by flow cytometry using a Guava Muse^®^ Cell Analyzer (EMD Millipore Cat. No 0500-3115, Serial No. 72001504335, Burlington, MA, USA). The ViaCount reagent distinguishes viable and non-viable cells based on the differential permeability of two DNA-binding dyes, providing the exact cell count of suspended cells and the percentage of viable cells. Muse^®^ 1.4.0 Analysis Software (EMD Millipore) was used to collect and examine the data. Cells treated with DMSO were used as controls. The percentage of viable (live) cells was represented in relation to the total cell number under each experimental condition. The cell number was represented as a percentage relative to the cell number in the control (100%).

### 2.4. Clonogenic Assay

During the exponential growth phase, the cells were trypsinized, and 300 single-cell suspensions were seeded in triplicate in complete medium in 6-well plates and incubated to adhere overnight at 37 °C under 5% CO_2_. The next day, the medium was replaced with DMSO control or drug-containing medium, and the cells were kept in an incubator at 37 °C for 9–11 days. After fixing with 10% formalin and staining with 0.05% crystal violet, colonies containing more than 50 cells were counted. The surviving fraction was normalized to the plating efficiency of the corresponding DMSO controls using the following formula: number of colonies after treatment/(number of cells plated × plating efficiency of DMSO-treated control cells). Plating efficiency of DMSO-treated control cells = (number of colonies formed in DMSO control/number of cells plated) × 100% [40]. Alternatively, to evaluate the residual toxicity of auranofin (chronic long-term effects) on GBM cells, 300 cells that were viable upon exposure to auranofin for 72 h were seeded in a 6-well plate containing drug-free medium and allowed to grow for 9–11 days until DMSO-treated control cells exhibited positive colonies (50 or more cells).

### 2.5. Western Blot Analysis

Cells were grown overnight in standard medium and treated (drug or control) for the indicated time and concentration. The cell protein extraction was performed as previously described [41]. Protein concentrations in the samples were determined using Pierce BCA Protein Assay Kit (Thermo Fisher Scientific Inc., Waltham, MA, USA). The Cytation 3 Multi-Mode Reader from BioTek (Agilent) was used to quantify the absorbance at 562 nm. A total of 25 μg of proteins per sample were subjected to electrophoretic separation using 10 or 12% SDS-PAGE (TGX Stain-Free FastCast Acrylamide kit, Cat. No. 1610183; 1610185, BioRad Laboratories Inc., Hercules, CA, USA) and subsequently transferred onto Immuno-Blot^®^ PVDF membranes (Cat. No.10026934, BioRad Laboratories Inc.) using a Trans-Blot^®^ Turbo™ Transfer System (Serial No. 690BR014594, BioRad Laboratories Inc.). Membranes were blocked with 5% milk at room temperature for 1 h followed by 5 washes of 5 min with 1X TBS-Tween and incubated at 4 °C overnight in primary antibodies including (Table S1) phosphorylated EGFR (p-EGFR/Y1068) (D7A5) XP^®^, total EGFR (1005), p-Akt/Ser473 (193H12), Akt, TrxR1 (B-2), NRF2 (D1Z9C), phospho-histone γH2A.X (Ser139), ubiquitin P37, β-Actin, and PARP. Corresponding goat anti-mouse IgG (H+L)-HRP conjugate (Cat. No. 170-6516, BioRad Laboratories Inc., 1:5000) or goat anti-rabbit IgG (H+L) conjugate (Cat. No. 170-6515, BioRad Laboratories Inc., 1:5000) horseradish peroxidase-conjugated secondary antibodies were used for 1 h of incubation followed by 5 washes of 5 min with 1X TBS-Tween. Protein detection was performed via a ChemiDoc Imaging System (Serial No. 732BR1945, BioRad Laboratories Inc.) using chemiluminescence Clarity Western ECL Imaging System (Cat. No. 170-5060, BioRad Laboratories Inc.). Using Image Lab™ Touch Software Version 1.2 (BioRad Laboratories Inc.), densitometric results were normalized to the control group actin.

### 2.6. Microscopy Fluorescence Imaging

To visualize ROS by microscopy, GBM cells were seeded in an 8-chamber cell culture slide (Cat. No. 229168, Ultident Scientific, Montreal, QC, Canada), incubated overnight, then treated with 3 and 6 μM auranofin for 24 h. Thereafter, the medium was replaced by red phenol-free media containing 5 μM of the general stress oxidative indicator CM-H2DCFDA, (Cat. No. C6827, Invitrogen) for 20 min, followed by 2 μM Hoechst 33342 dye (Cat. No. 62249, Thermo Scientific, Waltham, MA, USA) or 0.1 μg/mL DAPI (Cat. No. D9542, Sigma) used to stain nuclei for 10 min at 37 °C in 5% CO_2_. Wells were washed once and left in 1X PBS. Axio Vert.A1 microscope (Cat. No. 491206-0002-000, Zeiss, Oberkochen, Germany) and EVOS M5000 Imaging System microscope (Cat. No. 12563631, Invitrogen) were used to capture the images.

### 2.7. Annexin-V

U87MG cells were treated with 1, 3, or 5 μM auranofin for 72 h, collected and resuspended at 1 × 10^6^ cells in 100 μL. The suspension was stained with 100 μL of Muse^®^ Annexin V & Dead Cell Reagent (Cat. No. MCH100105, EMD Millipore Corp.) for 20 min at room temperature in the dark. Annexin V, a calcium-dependent phospholipid-binding protein, binds to phosphatidylserine, which translocates to the extracellular surface during apoptosis. The 7-AAD reagent is excluded from live healthy cells, while late apoptotic and dead cells are permeable. The cells (dead, live, early, and late apoptotic) were analyzed using the Guava Muse^®^ Cell Analyzer (EMD Millipore, Burlington, MA, USA).

### 2.8. Measurement of Intracellular General ROS and Superoxide Anions

To determine whether auranofin increased intracellular ROS generation in GBM cells, the cells were seeded in 96-well plates, incubated overnight, then treated with 3 and 6 μM auranofin alone or in combination with 5 μM L-BSO for 2.5 or 24 h; then, the medium was replaced by red phenol-free media containing 5 μM of the general stress oxidative indicator CM-H2DCFDA (Cat. No. C6827, Invitrogen) for 20 min followed by 2 μM Hoechst 33342 dye (Cat. No. 62249, Thermo Scientific, Waltham, MA, USA) used to stain nuclei for 10 min at 37 °C in 5% CO_2_. CM-H2DCFDA was measured at ex/em 493/528 nm. Wells were washed once and left in 1X PBS. For Hoechst 33342, the plates were measured at ex/em 350/450 nm, and values were used to normalize ROS.

The intracellular superoxide levels were measured using the Luminex oxidative stress kit (Cat. No. MCH100111, EMD Millipore Corp.), which includes dihydroethidium (DHE), a cell-permeable reagent that binds to DNA and produces red fluorescence as a readout of superoxide interaction with DNA. Cells were treated, incubated with DHE, collected, and processed in 1X assay buffer at a concentration of 1 × 10^6^ to 1 × 10^7^ cells per mL. The Muse oxidative stress reagent in 1X assay buffer was diluted to prepare the Muse oxidative stress working solution. Cells were incubated with the working solution at 37 °C for 30 min. Following incubation, the stained samples were analyzed using the oxidative stress protocol in the Muse cell analyzer (EMD Millipore Corp).

### 2.9. Combination Index Analysis

At a cell confluency of 70%, cells were seeded in triplicate in 96-well plates with 2.5 × 10^3^ cells/well in DMEM supplemented and incubated at 37 °C in 5% CO_2_. The cells were then treated with varying concentrations of auranofin (1–3 μM), either alone or in combination with L-BSO (5 μM), and allowed to grow for 72 h. Thereafter, the MTT assay was performed to determine vitality [38]. We used the CompuSyn software version 1.0 to calculate the combination index (CI) following the Chou–Talalay method [42] that denotes drug interaction.

### 2.10. Determination of TrxR Activity

TrxR activity was measured with the thioredoxin reductase colorimetric assay kit (Cat. No. ab83463; Abcam, Cambridge, MA, USA), as previously reported according to the manufacturer’s guidelines [41]. The assay principle consists of reducing 5,5′-dithiobis (2-nitrobenzoic) acid (DTNB) with NADPH to form TNB. Cells were homogenized on ice with 100 μL of cold 1X assay buffer containing 1X protease inhibitor cocktail (Cat. No. ab65621, Abcam) and 50 μg of protein was used for each analysis. One unit of TrxR was considered the amount of enzyme that generates 1.0 µmol of TNB per minute at 25 °C.

### 2.11. Glutathione (GSH) Assay

GSH levels were measured using a GSH assay colorimetric kit (Cat. No ab239727, Abcam) following the manufacturer’s instructions [41]. The kit contains a chromophore, and the reduction in the chromophore by an enzyme can be determined kinetically by measuring the absorbance at 450 nanometers. The absorbance is directly proportional to the reduced GSH present in each sample. The assays were carried out in 96-well microplates in duplicates. The rate of the background-corrected samples was calculated as follows: rate background corrected samples = [rate _sample_ − rate _background control_].

### 2.12. EGFR and GCLC Co-Expression Correlation in Brain Cancer and GBM Datasets

The correlation between the expression of *EGFR* and *GCLC* genes was processed as previously reported [43]. The RNA-sequencing data of *EGFR* and *GCLC* genes were retrieved from ARCHS4, a publicly accessible database of standardized RNA-Seq counts across human sequencing samples generated from the Illumina HiSeq 2000, HiSeq 2500, or NextSeq 500 platforms [44]. To specifically generate normal and brain cancer tissue correlation data, RNA-sequencing read counts were pre-processed with custom R scripts for their categorization, filtering, normalization, transformation, and the correlation calculation was performed in the correlation AnalyzeR R package https://gccri.bishop-lab.uthscsa.edu/shiny/correlation-analyzer/, (accessed on 10 March 2024). RNA-Seq Count data were filtered to remove samples with low total read counts (less than 5 million raw read counts) to reduce noise from low-quality samples. Normalization of count data was performed using DESeq2 normalization and a variance stabilizing transform. Genome-wide distribution of *EGFR* and *GCLC* correlation values (histogram) and scatter plots were generated comparing *EGFR* and *GCLC* expression across normal or brain cancer samples. The performance of Pearson and Spearman correlation methods were compared, and Pearson correlations with p-adjusted values from “Holm” correction were calculated using the cor function of the WGCNA R package, Licence: GNU GPL 3, version 1.19.

The correlation values of *EGFR* and *GCLC* genes were further analyzed using The Cancer Genome Atlas (TCGA, Firehose Legacy, https://www.cancer.gov/tcga, accessed on 10 March 2024). GBM patient dataset, sourced from cbioportal.com, accessed on 14 March 2024. Data for mRNA expression levels for *EGFR* and *GCLC* genes using the Affymetrix human genome U133 microarray platform were available for 528 GBM patients’ samples. Data were normalized using log2 transformation. For subsequent subgroup analysis, mRNA expression values of *EGFR* were categorized using “*EGFR* alterations” (mutations, amplification) vs. “*EGFR* no alterations” (wild-type *EGFR* gene), and the Pearson correlation coefficient between *EGFR* and *GCLC* genes was analyzed.

### 2.13. Statistical Analysis

We used GraphPad Prism version 8.0.2 (GraphPad Software Inc., La Jolla, CA, USA) for statistical analysis. Data are reported as mean ± SEM and are representative of at least 3 independent experiments unless otherwise stated. One-way ANOVA for analysis of one independent variable followed by Tukey post hoc multiple comparisons testing or two-way ANOVA for two independent variables were performed for comparisons involving three or more groups. Bonferroni post hoc testing was used for t-distribution multiple comparisons testing and Dunnett test was used to compare every mean to a control mean; *p* values < 0.05 were considered statistically significant.

## 3. Results

### 3.1. Auranofin-Induced Cytotoxicity in Both Parental and Isogenic EGFRwt or EGFRvIII GBM Cell Lines

**Figure 1:**
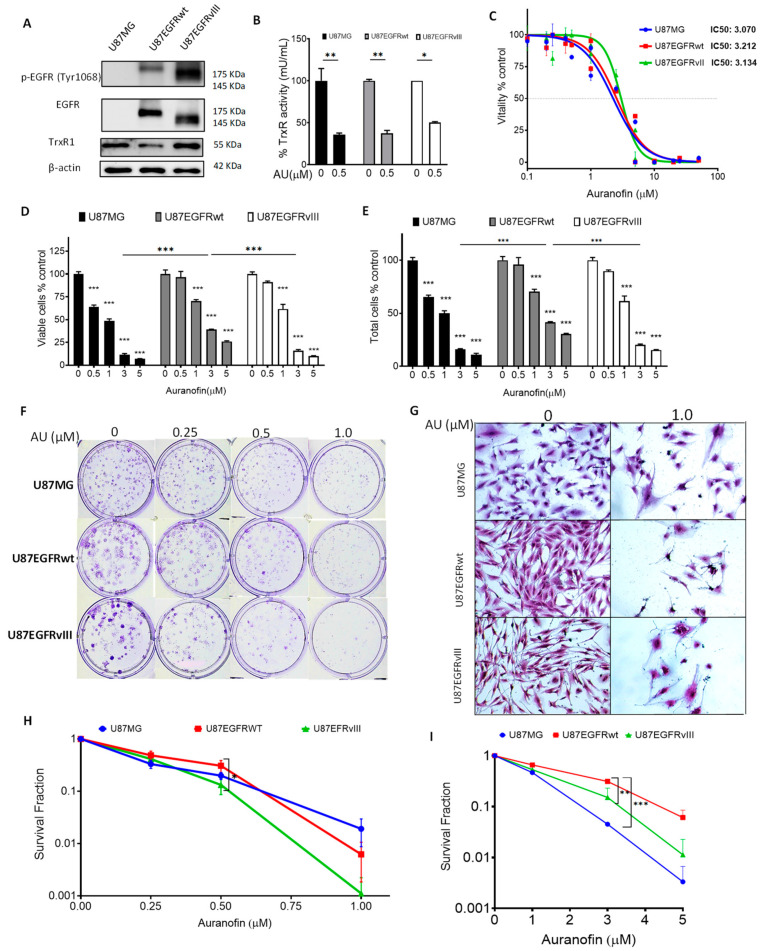
Auranofin (AU) cytotoxicity in U87/EGFR isogenic GBM cell lines. (**A**) Western blot analysis of phosphorylated-EGFR Tyr1068 (p-EGFR), total EGFR, and TrxR1 basal expression levels in U87MG cells, U87/EGFRwt, and U87/EGFRvIII GBM cell lines. β-actin was used as a loading control. (**B**) TrXR activity after treatment with 0.5 μM AU for 24 h compared to DMSO control. Error bars represent the standard error of the mean (SEM) (* *p* < 0.05, ** *p* < 0.01) (**C**) MTT assay showing the IC_50s_ of the EGFR isogenic cell lines in cells treated with AU for 72 h. (**D**) Viability and (**E**) total cell count of cells treated with AU for 72 h; bar charts show the mean ± SEM (* *p* < 0.05, ** *p* < 0.01, *** *p* < 0.001). (**F**) Clonogenic assay showing colony-formation capacity at different concentrations of AU after 9–10 days incubation. (**G**) Microscopic images of colonies in control (0 µM) and abortive colonies (<50 cells) following treatment with AU at 1 µM. (**H**) Survival fraction (Log scale) of U87MG, U87/EGFRwt, and U87/EGFRvIII cells; each data point shows the mean ± SEM; significant difference between U87/EGFRwt and U87/EGFRvIII at 0.5 µM AU (* *p* < 0.05). (**I**) Residual cytotoxicity of AU (Log scale): Colony formation capacity after treatment with AU for 72 h, followed by plating viable cells in drug-free media for 9–10 days. Each data point shows the mean ± SEM; significant difference between U87/EGFRwt and U87/EGFRvIII at 3.0 µM AU (* *p* < 0.05, ** *p* < 0.01); significant difference between U87/EGFRwt and U87MG at 3.0 µM AU (*** *p* < 0.001). Graphs represent the mean ± SEM from three independent experiments in triplicate.

Recent studies revealed the anticancer effects of auranofin in different cancer types [45], including GBM [46,47,48]. Aberrant EGFR expression is associated with increased oxidative stress secondary to EGFR hyper-activation [23,29]. To investigate the hypothesis that auranofin may affect the viability of GBM cells harboring aberrant EGFR expression, we used U87MG GBM cell lines isogenic for EGFR expression. We previously characterized U87MG and its counterpart stably transfected with EGFRwt (U87/EGFRwt) or EGFRvIII (U87/EGFRvIII) for their response to EGFR-targeting and DNA-damaging drugs [37]. First, we performed immunoblotting to assess Tyr1068 phosphorylation (p-EGFR) and expression levels of EGFRwt, EGFRvIII, and expression of TrxR1, the main target of auranofin [49,50]. As expected, U87MG cells did not show p-EGFR or EGFR expression. U87/EGFRvIII displayed higher p-EGFR expression compared to U87/EGFRwt. TrxR expression basal levels were nearly similar among the three cell lines (Figure 1A). Auranofin at only 0.5 µM for 24 h significantly decreased TrxR activity in U87MG cells (*p* = 0.003), U87/EGFRwt (*p* = 0.004), and U87/EGFRvIII cells (*p* = 0.025), compared to DMSO-treated controls. TrxR activity was not significantly different among the three cell lines in response to auranofin (Figure 1B).

We assessed the cytotoxicity of auranofin on U87/EGFR isogenic cell lines treated with different concentrations of auranofin for 72 h using an MTT assay to measure their vitality (i.e., cellular wellbeing) [38]. There was no statistically significant difference in the half-maximal inhibitory concentrations (IC_50s_) among the cell lines (Figure 1C, IC_50s_, 3 µM). Cell vitality using MTT measures metabolic activity, which may not be directly proportional to the actual cell number [51,52]. Hence, we further measured viability and total cell number by flow cytometry. Auranofin significantly decreased viability (Figure 1D) and the total number of cells (Figure 1E), compared to their respective controls in a dose-dependent manner. Interestingly, U87/EGFRvIII displayed similar sensitivity as U87MG and was significantly more sensitive to auranofin-induced decrease in total cell number (*p* < 0.001) and cell viability (*p* < 0.001) compared to U87EGFRwt cells (Figure 1E).

To investigate the long-term cytotoxic effects of auranofin, we performed a clonogenic survival assay to determine the capacity of the drug to inhibit colony formation in U87/EGFR isogenic cells. Auranofin induced long-lasting cytotoxic effects (9–10 days) at concentrations lower than the IC_50_ calculated by MTT (0.25 μM, 0.5 μM, and 1.0 μM, as seen in Figure 1F). Exposure to only 1.0 µM auranofin impeded colony formation and induced spindle-shaped cell morphology and scattered cells in abortive colonies in comparison to dense colonies in the DMSO control (Figure 1G). Auranofin significantly decreased the survival fraction of U87MG, U87/EGFRwt, and U87/EGFRvIII cells compared to their respective controls (*p* < 0.001 for the three cell lines). While there was no statistically significant difference between U87/EGFRvIII and U87MG (*p* > 0.05), auranofin at 0.5 μM significantly decreased U87/EGFRvIII clonogenic potential in comparison to U87/EGFRwt (*p* = 0.036) (Figure 1H).

We further analyzed the residual long-term cytotoxicity of auranofin, as readout of potential resistance to auranofin treatment. Cells were exposed to different concentrations of auranofin or DMSO control for 72 h and viable cells were seeded for a colony-formation assay in drug-free medium. Over the course of 9–10 days, the percentage of positive colonies relative to control decreased in a dose-dependent manner. Interestingly, auranofin at 3 μM decreased the clonogenic ability of U87/EGFRvIII and U87MG cells to the same extent (*p* > 0.05), while their sensitivity was significantly higher compared to that of the U87/EGFRwt cell line (*p* = 0.002) (Figure 1I and Figure S1AB). Overall, auranofin induced short- and long-term cytotoxicity in both U87MG and isogenic EGFRwt, EGFRvIII cell lines. U87/EGFRvIII showed heightened sensitivity to auranofin, i.e., significant decrease in viability, cell number, clonogenicity (at 0.5 μM), and residual long-term cytotoxicity compared to U87/EGFRwt cells.

### 3.2. Auranofin Increased ROS along with ROS-Dependent Cytotoxicity and Molecular Effects in GBM U87/EGFR Isogenic Cell Lines

**Figure 2:**
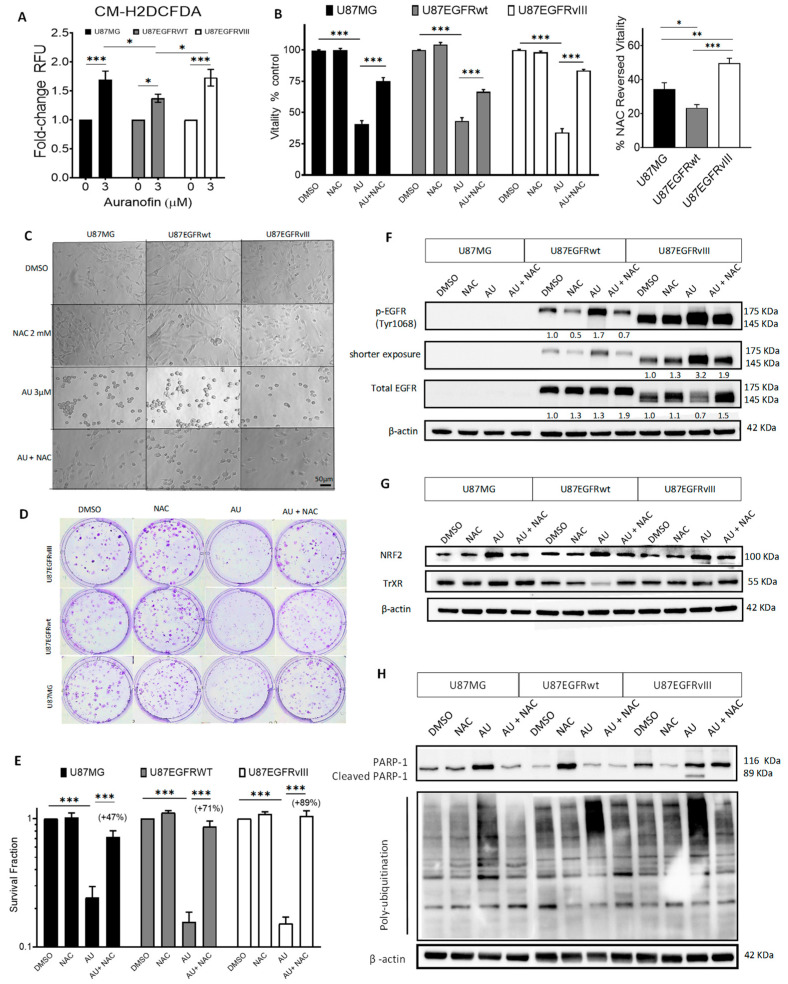
Auranofin (AU)-induced ROS: Cytotoxic and molecular ROS-dependent effects in U87/EGFR isogenic cell lines. (**A**) Fold change of relative fluorescence units (RFU) of ROS intracellular levels in U87MG, U87/EGFRwt, and U87/EGFRvIII cells using a microplate reader (AU, 3 µM, 24 h). Error bars represent the standard error of the mean (SEM) (* *p* < 0.05, *** *p* < 0.001). (**B**) MTT assay showing the effect of AU alone or in combination with N-acetylcysteine (NAC) on cell vitality (**left**); bars show the mean ± SEM (*** *p* < 0.001). NAC protection effects on vitality relative to AU-treated cells (**right**); bars show the mean ± SEM (* *p* < 0.05, ** *p* < 0.01, and *** *p* < 0.001). (**C**) Phase contrast imaging of cell morphology upon treatment with AU or in combination with NAC. (**D**) Colony-formation assay plates representative of Figure 2G; (6× magnification). (**E**) Clonogenic assay showing surviving fraction (Log Scale) following treatment with AU 0.5 μM alone or in combination with NAC, 2 mM for 9–11 days; NAC protective effect against decreased clonogenic survival is expressed as a percentage relative to AU-treated cells. Bars show the mean ± SEM (*** *p* < 0.001). Graphs represent mean values ± SEM from at least three independent experiments in triplicate. (**F**) Western blotting analysis showing the impact of auranofin alone (3 µM, 24 h) or in presence of NAC 2 mM on phosphorylated EGFR, total EGFR, (**G**), NRF2, TrxR1 (**H**), PARP-1 cleavage, and protein polyubiquitination in the three cell lines. DMSO was used as a vehicle control and actin as a loading control. “Shorter exposure” refers to a reduced time exposure to avoid signal saturation for U87/EGFRvIII.

Auranofin increases the production of ROS and disrupts intracellular redox homeostasis in various cancer cells [48,53,54,55,56,57]. Using the fluorescent general ROS-sensitive probe CM-H2DCFDA, we assessed auranofin-induced ROS levels in U87/EGFR isogenic cell lines by fluorescence. Auranofin treatment at the IC_50_, 3 µM for 2.5 h did not significantly increase ROS, while significant levels of ROS were induced only at a higher dose (6 µM for 2.5 h) in the three cell lines (Figure S2A). Treatment with auranofin at 3 µM for 24 h significantly increased ROS levels in the three isogenic cell lines compared to their respective controls (U87MG *p* < 0.001, U87EGFRwt, *p* = 0.024, U87EGFRvIII *p* < 0.001) (Figure 2A). Of note, ROS increase was more pronounced in U87/EGFRvIII compared to U87/EGFRwt (*p* = 0.033), as shown in Figure 2A, and illustrated by fluorescence microscopy (Figure S3B).

Next, we used N-acetylcysteine (NAC), a conventional ROS scavenger [47,58], to determine whether ROS mediate the cytotoxic effects of auranofin in U87/EGFR isogenic cell lines. NAC treatment at 1 or 2 mM for 72 h did not significantly affect cellular metabolic activity or vitality (Figure S3A). Fluorescence microscopy using the general ROS-sensitive probe CM-H2DCFDA illustrates increased intracellular ROS levels following auranofin treatment at 3 μM for 24 h, while the addition of 2 mM NAC prevented this increase in ROS in all cell lines (Figure S4). Vitality assay using 3 μM auranofin in the presence or absence of 2 mM NAC for 72 h showed that NAC significantly prevented the cytotoxic effects of auranofin on the three cell lines (34% reduction in U87MG, 22% in U87/EGFRwt, and 50% in U87EGFRvIII all *p* < 0.001) (Figure 2B, left). This effect was significantly more evident for U87EGFRvIII compared to U87MG (*p* = 0.003) and U87/EGFRwt (*p* < 0.001) (Figure 2B, right). Furthermore, NAC rescued auranofin-induced cell rounding, as shown by phase contrast microscopy (Figure 2C). The efficacy of NAC in preventing auranofin-decreased viability was validated in U87MG cells by flow cytometry analysis (Figure S3b).

NAC at 2mM significantly counteracted the cytotoxicity of 0.5 μM auranofin on colony formation in the three cell lines (*p* < 0.001) compared to auranofin treatment alone or DMSO control (Figure 2D). Notably, NAC prevented the decline of survival fractions in response to auranofin treatment 48% in U87MG, 71% in U87EGFRwt, and 89% in U87/EGFRvIII. This protective effect was significantly more pronounced in U87/EGFRvIII compared to U87MG (*p* = 0.018) but was not significant in comparison to U87EGFRwt cells (*p* = 0.4) (Figure 2E).

Previous studies reported the redox regulation of EGFR and the crosstalk of ROS with EGFRwt or EGFRvIII and their downstream-induced signaling [23,24,25,26,27]. We investigated the impact of EGFRwt or EGFRvIII overexpression on auranofin-induced molecular effects and the role of ROS in these effects by Western blotting in U87/EGFR isogenic cell lines. Auranofin treatment (IC_50_ 3 μM, 24 h) increased phospho-EGFR (Tyr1068), one of the major autophosphorylation sites of EGFR [59], compared to DMSO control in U87/EGFRwt and U87/EGFRvIII. Due to the high basal phosphorylation and subsequent oversaturation of chemiluminescence signal in U87/EGFRvIII, we show shorter exposure displaying marked increase in phospho-EGFR (Tyr1068) in U87EGFRvIII, compared to U87/EGFRwt. Strikingly, auranofin decreased the expression of total EGFR in U87EGFRvIII, but not in the U87/EGFRwt cell line. Of note, NAC at 2 mM prevented the upregulation of phospho-EGFR (Tyr1068) in U87EGFRwt and U87EGFRvIII, and the downregulation of total EGFR in U87EGFRvIII (Figure 2F). Densitometric analysis confirmed the differential effects of auranofin in U87/EGFRvIII vs. U87/EGFRwt. The ratio of phospho-EGFR (Tyr1068)/total EGFR increased by 4-fold in U87EGFRvIII compared to DMSO control, and treatment with 2 mM NAC completely maintained the ratio to basal levels (Figure S5).

Auranofin treatment decreased TrxR1 expression only in U87/EGFRwt, and this decrease was prevented by NAC (Figure 2G). Nuclear factor erythroid 2–related factor 2 (NRF2) is a redox-sensitive transcription factor that controls cellular Trx and GSH antioxidant response [60,61]. Following treatment with 3 µM auranofin for 24 h, Nrf2 expression was increased in U87MG, U87EGFRwt, and U87EGFRvIII cell lines. This upregulation was prevented by 2 mM NAC (Figure 2G).

Auranofin treatment (3 μM, 24 h) induced cleavage of poly(ADP-ribose) polymerase (PARP-1) a hallmark of apoptosis only in U87EGFRvIII cells displaying cleaved PARP-1 fragment (89 kDa), while this effect was prevented in the presence of 2 mM NAC (Figure 2H). Flow cytometry analysis of U87MG GBM cell line treated with DMSO vehicle control or auranofin (0–5 µM) for 72 h demonstrated that auranofin at 1 µM caused a significant increase in early stage apoptotic Annexin V-positive/7AAD-negative cells (Figure S6A). Meanwhile, auranofin at 3 and 5 µM for 72 h significantly increased late-stage apoptotic Annexin V/7AAD-positive cells (Figure S6B).

The anticancer effects of auranofin have been related to inhibition of deubiquitinases involved in proteasome-mediated protein degradation [62,63,64,65]. Anti-ubiquitin immunoblotting analysis showed evidence of increased polyubiquitination of proteins in the three GBM cell lines treated with auranofin (3 µM, 24 h), while this effect was prevented by NAC at 2 mM (Figure 2H).

### 3.3. EGFR Alterations Are Associated with Reduced GSH in GBM Cell Lines and Decreased Correlation between EGFR and GCLC Expression in Patient Datasets

**Figure 3:**
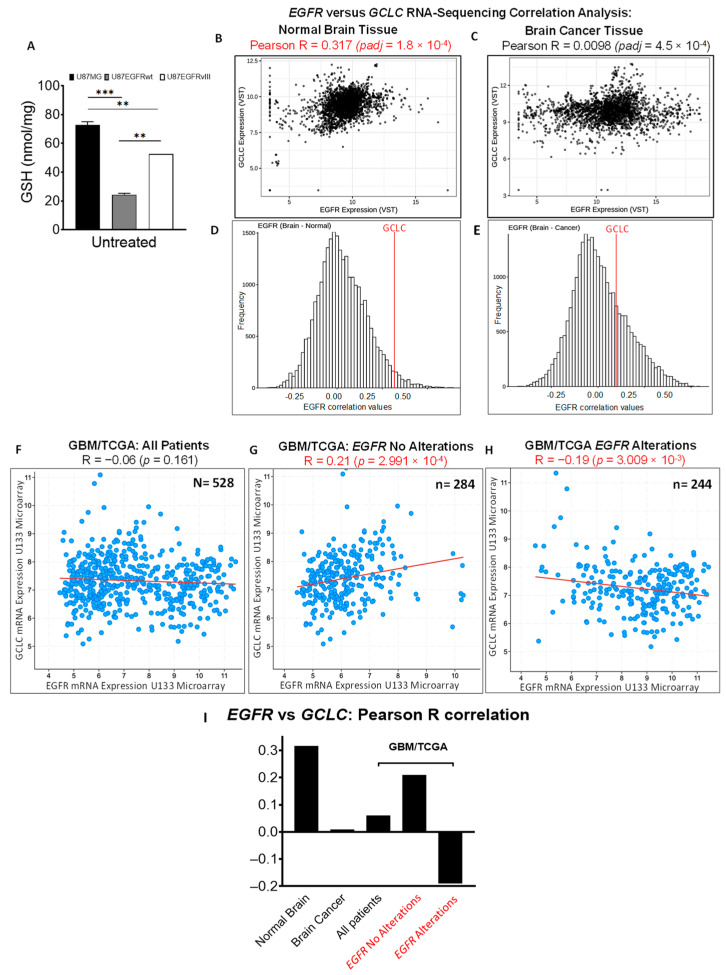
Inverse relationship between *EGFR* alterations and glutathione (GSH) levels in GBM cell lines and between *EGFR* and *GCLC* in patients’ datasets. (**A**) Basal GSH levels in U87MG, U87EGFRwt, and U87EGFRvIII cells; bars show the mean ± SEM (** *p* < 0.01 and *** *p* < 0.001). (**B**–**E**) Co-expression correlation analysis of EGFR and GCLC RNA-sequencing read counts using the Correlation AnalyzeR web application (Gene vs. gene mode, ARCHS4 data source). (**B**) Scatter plots visualize the correlation between co-expression of EGFR and GCLC (RNA-sequencing read counts), in samples from human normal brain tissue (N = 3198), but not in (**C**) brain cancer tissue (N = 2750). (**D**) Histogram analysis of genome-wide EGFR correlation values in normal brain tissue vs. (**E**) brain cancer tissue. The red line depicts the position of *GCLC* correlation value with *EGFR.* (**F**) Correlation analysis of *EGFR* and *GCLC* mRNA expression using all patients (N = 528) and subgroup analysis based on *EGFR* status. Positive correlation for (**G**) subgroup “no alterations” (N = 284), vs. a negative correlation for (**H**) subgroup “alterations” (*EGFR* amplification, mutations, N = 244) in The Cancer Genome Atlas Network (TCGA) GBM patient dataset. Pearson correlations with related *p*-values are shown for Correlation AnalyzeR and TCGA datasets (*p* < 0.05 indicates significance). (**I**) Graph summarizing Pearson correlations for EGFR and GCLC in Correlation AnalyzeR and TCGA analysis.

Increased basal levels of ROS in EGFR-driven GBM with EGFRvIII expression [29] and *EGFR* amplification [30] suggest a potential relationship between *EGFR* alterations and basal GSH antioxidant capacity in GBM. To investigate the relationship between *EGFR* alterations and GSH levels, we measured the intracellular basal levels of reduced GSH [66,67] in U87/EGFR isogenic cell lines. U87/EGFRwt cells showed significantly lower basal levels of reduced GSH compared to U87MG (*p* < 0.001) or U87/EGFRvIII cells (*p* = 0.004). Likewise, U87EGFRvIII showed significantly lower GSH basal levels compared to U87MG cells (*p* = 0.001) (Figure 3A). Lower intracellular GSH in U87/EGFRwt and U87/EGFRvIII compared to the U87MG cell line prompted our interest to investigate the relationship between expression of *EGFR* and *GCLC*, the key catalytic enzyme subunit for GSH biosynthesis [19].

We performed computational analysis of *EGFR* and *GCLC* expression and their correlation analysis using publicly available RNA-sequencing datasets [43]. To gain new insights into the impact of disease on co-expression of *EGFR* and *GCLC* genes, we analyzed the correlation between *EGFR* and *GCLC* co-expression in human normal brain and brain cancer tissues (Figure 3B–E). As expected, scatter plot analysis of *EGFR* and *GCLC* co-expression showed that the distribution of EGFR tissue expression in normal brain samples (N = 3198, Figure 3B) shifts toward higher expression levels in brain cancer tissue (N = 2750, Figure 3C). *EGFR* and *GCLC* displayed a significant positive correlation in normal brain tissue (Pearson R = 0.317, *padj* = 1.8 × 10^−4^), which strikingly decreased to a near-zero value in brain cancer (Pearson R = 0.0098, *padj* = 4.5 × 10^−4^). Histogram analysis of genome-wide EGFR correlation values integrating the position of *GCLC* gene highlights the shift of *GCLC* and *EGFR* correlation value in normal brain tissue (Figure 3D) vs. cancer tissue (Figure 3E).

Next, we investigated the correlation between *GCLC* and *EGFR* expression in a dataset of patients exclusively diagnosed with GBM. We analyzed *EGFR* and *GCLC* mRNA expression in a cohort of GBM patients from the TCGA GBM patient dataset. There was no correlation between *EGFR* and *GCLC* mRNA expression in GBM across the complete patient dataset of tissue samples (N = 528, Pearson R= −0.06, *p* = 0.161, Figure 3F). To dissect whether *EGFR* alterations (amplification, mutations) affect its correlation with *GCLC*, we stratified the patient cohort into two groups based on *EGFR* status (alterations vs. no alterations). As previously reported [7], the distribution of patients’ samples with *EGFR* “no alterations” (Figure 3G) showed a shift toward predominant *EGFR* overexpression levels for *EGFR* alterations (Figure 3H). Interestingly, subgroup analysis of GBM patients revealed a significant positive correlation between *EGFR* “no alterations” and *GCLC* mRNA levels (n = 284, Pearson R = 0.21, *p* = 2.99 × 10^−4^). Conversely, there was a significant negative correlation between *EGFR* alterations and *GCLC* mRNA expression, (n = 244, Pearson correlation= −0.19, *p* = 3.00 × 10^−3^), as shown in Figure 3H. Thus, in accordance with relatively low intracellular GSH in U87/EGFRwt and U87/EGFRvIII compared to the U87MG cell line, *EGFR* and *GCLC* co-expression showed a significant decrease in EGFR-overexpressing brain cancer tissue compared to normal brain tissue. These results align with the significant decrease in *EGFR* and *GCLC* co-expression in the subgroup of GBM/TCGA patients with *EGFR* alterations, and predominant *EGFR* overexpression. Hence, low intracellular GSH in U87/EGFRwt and U87/EGFRvIII compared to the U87MG cell line and the relatively low basal expression of *GCLC* in patients with *EGFR* alterations suggest the potential to effectively target GCLC and decrease compensatory antioxidant GSH activity in this subgroup.

### 3.4. Co-Targeting TrxR and GCLC in U87/EGFR Isogenic Cell Lines Induced Synergistic Cytotoxicity Associated with Intracellular GSH Decrease

**Figure 4:**
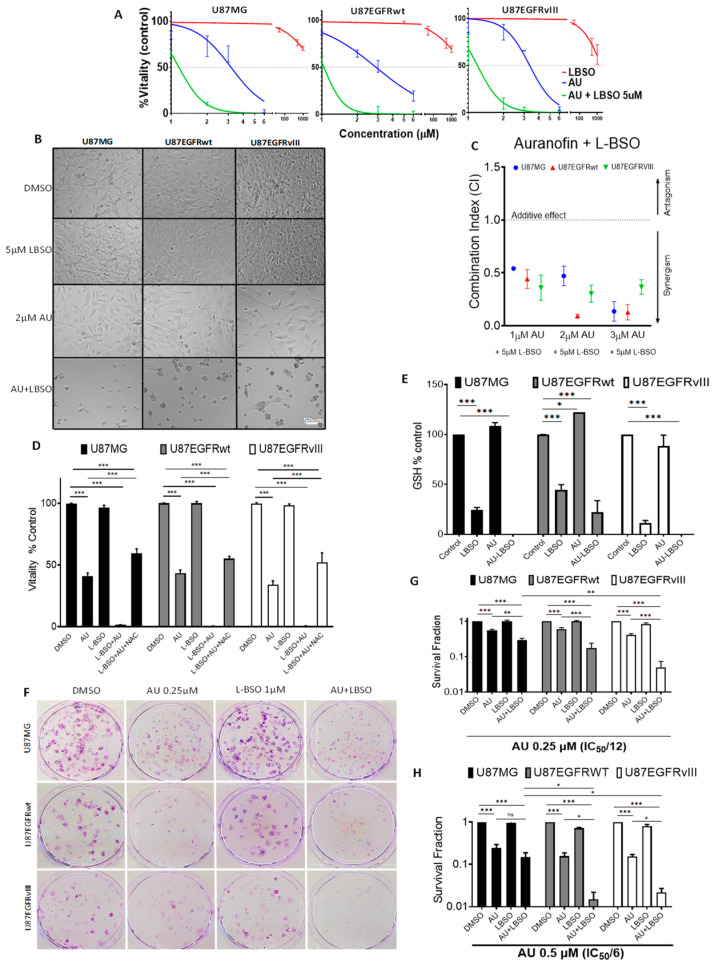
Impact of auranofin (AU) in combination with L-BSO on the survival and intracellular GSH of U87/EGFR isogenic cell lines. (**A**) U87MG, U87/EGFRwt, and U87/EGFRvIII cells were treated with different concentrations of AU alone or in combination with 5 µM L-BSO for 72 h; vitality was measured by MTT assay. (**B**) Representative images showing the cell morphology for 2 μM AU, 5 μM L-BSO, and their combination. (**C**) Combination index [CI] analyzed by the method of Chou–Talalay for drug combination analysis. Synergism was determined when CI was <1, antagonism when CI > 1, or additive effect when CI = 1. The values are shown for each drug concentration. (**D**) Effect on vitality measured by MTT assay using 2 mM NAC in cells treated with 5 µM L-BSO and 3 µM AU for 72 h in the three cell lines, bars show the mean ± SEM (*** *p* < 0.001). (**E**) GSH levels were quantified in GBM cells treated with 2 µM AU alone or combined with 5 µM L-BSO for 24 h, bars show the mean ± SEM (* *p* < 0.05, *** *p* < 0.001). (**F**) Colony-formation assay using 0.25 µM AU (IC50/12), 1 μM LBSO, or a combination of both. (**G**) Survival fraction (Log scale) calculated for 0.25 µM AU, 1 μM LBSO, or their combination, bars show the mean ± SEM (** *p* < 0.01, *** *p* < 0.001). (**H**) Survival fraction (Log scale) calculated for 0.5 µM AU, 1 μM LBSO, or their combination, bars show the mean ± SEM (* *p* < 0.05, *** *p* < 0.001).

Auranofin displays anticancer properties against various types of cancer cells, though its efficacy as a monotherapy cancer treatment is fairly limited in vivo [68]. L-BSO inhibits GCLC, leading to decreased compensatory GSH antioxidant activity [69,70,71]. We evaluated the interactions between auranofin and L-BSO in U87/EGFR isogenic cell lines. The MTT assay showed that L-BSO at 2 or 10 μM for 72 h did not affect the cellular vitality of the U87MG, U87EGFRwt, and U87EGFRvIII cell lines (Figure S7). While L-BSO at 5 μM did not significantly affect cell vitality, 5 μM L-BSO in combination with 1, 2, or 3 μM auranofin for 72 h significantly decreased the vitality of the three cell lines compared to auranofin treatment alone (*p* < 0.001) (Figure 4A). Figure 4B also illustrates the drastic morphological changes caused by 2 μM auranofin when combined with the non-toxic 5 μM L-BSO, such as increased cell rounding, detachment, and decreased cell density. The effects of other combined treatment conditions among these drugs on cell morphology are displayed in Figure S8. Drug interaction analysis showed that the addition of 5 μM L-BSO to 1, 2, or 3 μM auranofin was synergistic (Combination Index; CI < 1) in all GBM cell lines (Figure 4C and Table S2). Interestingly, the addition of 2 mM NAC to the combined treatment of 3 μM auranofin and 5 μM L-BSO prevented the reduction in the vitality by 58% in U87MG, 54% in U87/EGFRwt, and 51% in U87EGFRvIII (Figure 4D).

Previous studies reported GSH-induced compensation mechanisms in response to TrxR1 inhibition [19,21,72,73,74,75]. Following 24 h exposure to 2 µM auranofin, GSH levels increased significantly in U87EGFRwt cells (*p* = 0.045), compared to the DMSO control group, while levels of GSH in U87MG and U87EGFRvIII cells were unaffected (*p* > 0.05). L-BSO at 5 µM significantly decreased GSH levels (*p* < 0.001) in the three cell lines, compared to their respective DMSO control group. The combination of auranofin and L-BSO significantly depleted GSH levels in comparison to the DMSO control group (*p* < 0.001) in all cell lines (Figure 4E).

We further investigated whether the combination induced synergistic long-term effects. Clonogenic assay revealed that the combination of L-BSO at only 1 μM with auranofin at 0.25 μM, a concentration twelve-fold lower than the IC_50_, further significantly decreased the clonogenicity of U87MG by 1.86-fold (*p* = 0.003), U87/EGFRwt by 3.45-fold (*p* < 0.001), and U87/EGFRwt by 8.30-fold (*p* < 0.001), compared to auranofin alone. There was no significant difference between U87/EGFRVIII and U87/EGFRwt (*p* = 0.169), while U87/EGFRVIII showed a significantly lower surviving fraction compared to U87MG (*p* = 0.002) (Figure 4F–G). Auranofin at 0.5 μM (six-fold lower than the IC_50_) combined with L-BSO at 1 μM decreased clonogenic survival by 1.65-fold in U87MG (*p* = 0.195), 10.42-fold (*p* = 0.014) in U87/EGRwt, and 7.11-fold (*p* = 0.026) in U87/EGFRvIII compared to auranofin alone (Figure 4H). Comparing auranofin-L-BSO combination among cell lines showed that U87EGFRwt (*p* = 0.027) and U87/EGFRvIII (*p* = 0.038) were significantly more sensitive compared to U87MG, while no significant difference was found between U87/EGFRVIII and U87/EGFRwt cell lines (*p* = 0.999). We did not test a combination of auranofin at 1 μM and L-BSO at 1 μM, due to the drastic suppression of clonogenic formation (~2 log units) following treatment with auranofin alone at 1 μM for the three cell lines (Figure 1H).

### 3.5. Auranofin and L-BSO Combination Synergistically Increased ROS-Dependent Cytotoxicity and Induced Differential Molecular Effects in U87/EGFR Isogenic Cell Lines

**Figure 5:**
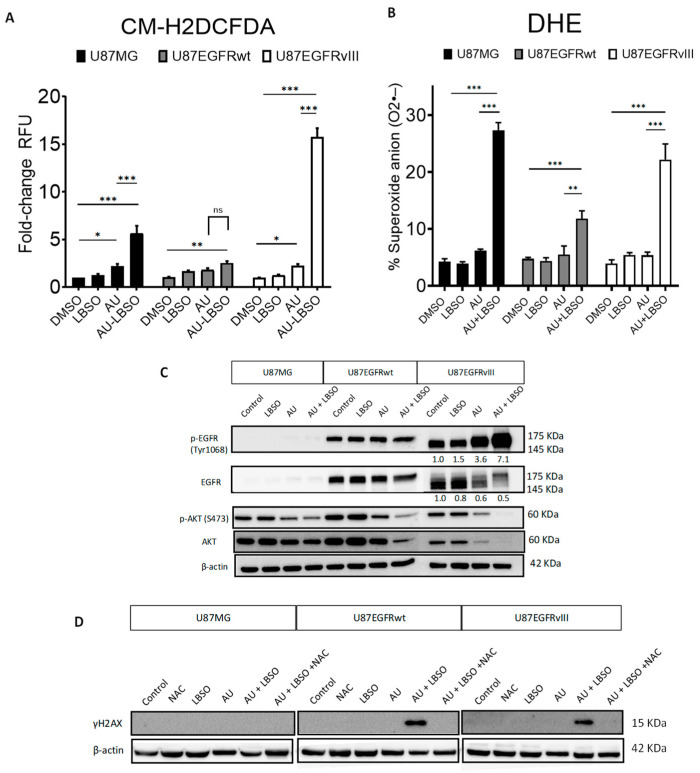
Synergistic effects of auranofin and L-BSO combination on ROS generation, EGFR signaling, and DNA damage. (**A**) U87MG, U87/EGFRwt, and U87/EGFRvIII cells were treated with 3 µM AU and 5 µM L-BSO for 24 h and ROS levels were quantified using the ROS indicator, CM-H2DCFDA; bars show the mean ± SEM (ns: not significant, * *p* < 0.05, ** *p* < 0.01, *** *p* < 0.001) or (**B**) dihydroetidium (DHE) was used to detect superoxide radical anion; bars show the mean ± SEM (** *p* < 0.01, *** *p* < 0.001) (**C**) U87MG, U87/EGFRwt, and U87/EGFRvIII cells were treated with 3 µM AU alone or in combination with 5 µM L-BSO for 6 h and phosphorylated and Total EGFR, and AKT expression was assessed by Western blotting. (**D**) Expression of the DNA damage marker γH2AX in GBM cells treated with 2 µM AU alone or in combination with 5 µM L-BSO, with or without NAC, 2 mM for 24 h.

We assessed the effect of auranofin and/or L-BSO combined treatment on intracellular general ROS levels, compared to each treatment alone. U87MG, U87/EGFRwt, and U87/EGFRvIII cells were treated with 3 µM auranofin, alone or in combination with 5 μM L-BSO for 24 h. We observed higher intracellular general ROS levels in the cells when the drugs were combined, in comparison to their DMSO control (U87MG *p* < 0.001, U87/EGFRwt *p* < 0.01, and U87/EGFRvIII *p* < 0.001). Of note, auranofin/L-BSO combined treatment induced the highest ROS increase in U87/EGFRvIII (15.7-fold ± 0.91) compared to U87MG (5.6-fold ± 0.76) or U87/EGFRwt (2.5-fold ± 0.19), as shown in Figure 5A.

We further evaluated the effect of auranofin/L-BSO combined treatment on superoxide anion generation in U87MG, U87EGFRwt, and U87EGFRvIII cells. Auranofin at 3 μM, combined with 5 μM L-BSO for 24 h, resulted in greater superoxide generation compared to their DMSO controls. Auranofin/L-BSO induced higher superoxide levels in U87MG cells when compared to U87EGFRwt cells (*p* < 0.001) or U87/EGFRvIII cells (*p* < 0.01) (Figure 5B).

Next, we used Western blotting to analyze the molecular effects of auranofin/L-BSO combined treatment in U87/EGFR isogenic cell lines treated with 3 μM auranofin alone, 5 μM L-BSO alone, or their combination for 6 h. As expected, there was no expression of p-EGFR (Tyr1068) or total EGFR in U87MG cells. Auranofin, L-BSO, or their combination did not affect EGFR phosphorylation (Tyr1068) or total EGFRwt expression in U87/EGFRwt cells. In sharp contrast with U87/EGFRwt, auranofin increased the phosphorylation of p-EGFR (Tyr1068) in U87EGFRvIII cells, which was further substantially increased following treatment with auranofin/L-BSO combination. Remarkably, these effects were associated with decreased expression of total EGFRvIII. The Ras/PI3K/AKT pathway is one of the major pathways that regulate cell proliferation, survival, and differentiation downstream of EGFR signaling [76]. Auranofin treatment decreased the phosphorylation of p-AKT(Ser473) in the three cell lines. Auranofin and L-BSO combination further decreased p-AKT(Ser473), and total AKT levels in U87/EGFRwt and to more pronounced undetectable levels in the U87EGFRvIII cell line (Figure 5C). Cells treated with a combination of 2 μM auranofin and 5 μM L-BSO for 24 h exhibited high phosphorylation levels of the DNA damage double-strand break marker γH2AX (Ser139) in U87/EGFRwt cells and U87EGFRvIII cells, but not in U87MG cells, while such effect was abrogated by 2 mM NAC (Figure 5D).

## 4. Discussion

**Figure 6:**
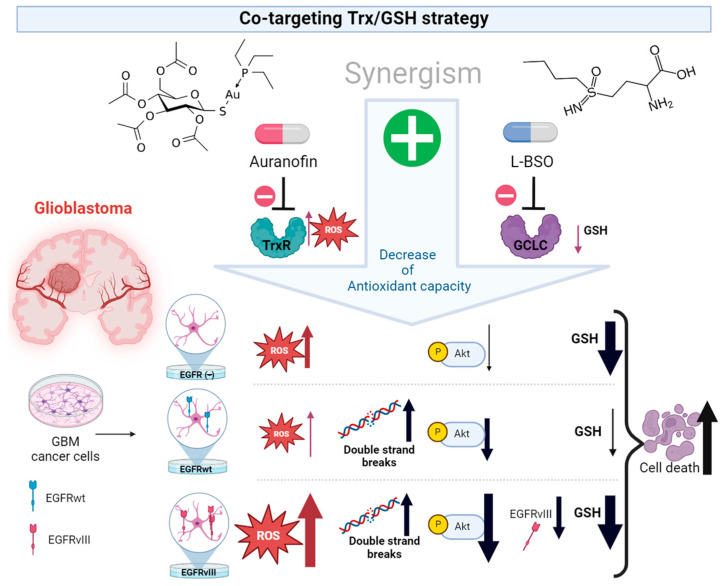
Model for synergistic prooxidant auranofin and L-buthionine–sulfoximine (L-BSO) combination to kill glioblastoma (GBM) cells, derived from studies on U87MG, U87/EGFRwt, and U87/EGFRvIII isogenic GBM cell lines. Auranofin targets thioredoxin reductase 1 (TrxR1) and L-BSO targets glutamate–cysteine ligase catalytic (GCLC) subunit required for glutathione (GSH) biosynthesis to increase cellular levels of damaging reactive oxygen species (ROS). Auranofin, but not L-BSO, induces cytotoxicity in the three cell lines. The combination of auranofin and L-BSO leads to synergistic cytotoxicity associated with the depletion of intracellular GSH. ROS increase, DNA damage, downregulation of phospho-(P)AKT(Ser473), and total AKT expression were induced to a differential extent based on EGFR expression levels. The most prominent decrease in P-AKT(Ser473) and total AKT is associated with EGFRvIII downregulation in the U87/EGFRvIII cell line.

Sustained ROS production is a hallmark of cancer cells, including cells representing GBM. Pro-oxidant strategies targeting redox homeostasis fail to reach the lethal ROS threshold in GBM cancer cells with different molecular profiles within highly heterogeneous GBM tumors. In this study, we exploited the dependency of EGFR-positive cells from their antioxidant systems as a vulnerability to effectively target their redox homeostasis. Using GBM cell lines isogenic for EGFRwt and EGFRvIII, we unraveled novel findings related to the impact of EGFR alterations on the response to auranofin or the combination of auranofin with L-BSO as pro-oxidant strategies (Figure 6).

Auranofin-induced cancer cell death has been under scrutiny since 2010 [77]. In this study, auranofin decreased the vitality and long-term clonogenic capacity at concentrations lower than the IC_50_ in the three cell lines compared with their respective controls. The ROS-scavenger NAC [58] prevented these cytotoxic effects, suggesting that they were mediated through ROS-dependent mechanisms. Comparing the response of the isogenic cell lines to auranofin revealed the cellular and molecular impact of EGFR alterations. Several lines of evidence suggest heightened sensitivity of U87/EGFRvIII to auranofin compared to U87/EGFRwt: (i) significant decrease in the total cell number and viability at the IC_50_ 3 µM and at 5 µM; (ii) decreased survival fraction compared to U87/EGFRwt cells at 0.5 μM auranofin; (iii) NAC prevented auranofin-induced cytotoxicity to a significantly higher extent in U87EGFRvIII compared to U87/EGFRwt or U87MG cells, suggesting that U87/EGFRvIII cells rely more on ROS-dependent mechanism for their survival. Auranofin-induced cytotoxicity was associated with molecular effects modulated to a differential extent in U87/EGFRvIII, compared to U87/EGFRwt or U87MG cell lines. These effects include ROS increase, PARP-1 cleavage, increased EGFRvIII/Tyr1045 phosphorylation, pronounced downregulation of total EGFRvIII, p-Akt, and total Akt.

Auranofin significantly and equally inhibited its primary target TrxR1 in both parental and EGFR-transfected cells at a concentration six-fold lower than their IC_50_. It is worth noting that the inhibition of the activity of TrxR1 alone may not be sufficient for killing GBM cells, as evidenced by the fact that auranofin did not lead to cell death at the low concentration that inhibited TrxR1. These results are consistent with those of Van Loenhout [48] reporting TrxR inhibition with sublethal auranofin concentrations and the potential role of other mechanisms in its cytotoxicity in U87MG. Additional mechanisms of auranofin, such as proteasome inhibition, may contribute to the observed cytotoxicity [78].

Compounds that inhibit TrxR enzymatic activity increase oxidative stress, shifting the cellular redox balance toward a more oxidized status [79,80]. Auranofin at the IC_50_ of 3 µM for 24 h significantly increased ROS levels in the three cell lines compared to their respective controls. Increased ROS production mediates cytotoxic cellular oxidative stress through impaired mitochondrial oxidative phosphorylation (OXPHOS), a crucial process for hydrogen oxidation to generate water and ATP. Auranofin forms within its gold(I) center a stable adduct with the selenocysteine residue in the active site of TrxR, leading to the inhibition of its catalytic activity and cellular oxidative stress [81]. A previous study showed that auranofin does not affect H_2_O_2_ formation by the mitochondrial respiratory chain but rather impairs H_2_O_2_ removal. TrxR inhibition increases H_2_O_2_ flow leading to a more oxidized mitochondrial environment, which is detrimental for mitochondrial functions [82].

Investigating the impact of auranofin on EGFR-induced signaling showed that auranofin induced the ROS-dependent downregulation of EGFRvIII and Akt, two critical oncogenic signaling molecules involved in cell proliferation and survival [76,83]. EGFR-induced activation of PI3K/Akt pathway plays a key role in GBM cancer cell survival [76,84,85]. The primary function of Akt activation to control energy metabolism is coupled to inhibition of apoptosis, while uncontrolled cell cycle progression is promoted. Inhibition of the EGFR/Akt pathway inhibits mitochondrial function and decreases the expression of lipid metabolism-associated genes, leading to decreased ATP production [84]. Conversely, the hyperactivation of EGFR/Akt activates NF-κB-dependent transcriptional expression of lipid metabolism-associated genes in the U87/EGFRvIII cell line and a primary patient-derived EGFRvIII mutant GBM cell line [85]. Thus, it is likely that auranofin triggers cell death by reducing the activity of the EGFRvIII/Akt pathway, lowering lipid metabolism, and decreasing ATP production.

EGFRvIII-positive cells showed greater vulnerability to EGFR downregulation in response to auranofin and auranofin/L-BSO treatment. Only a few compounds have been shown to increase ROS and induce EGFR downregulation through a degradation mechanism without directly targeting EGFR. Sanguinarine increases NOX3, leading to ROS increase, EGFR oxidation and degradation [86], curcumin induces the ubiquitin–proteasomal pathway with subsequent EGFR degradation [87], and arsenic induces autophagic EGFR degradation [88]. While auranofin and auranofin/L-BSO may induce one of the above mechanisms for EGFRvIII downregulation, we cannot exclude the potential role of EGFR Tyr-1068 phosphorylation, a major auto-phosphorylation site in EGFRvIII [89]. Auranofin and auranofin/L-BSO treatment increased EGFR Tyr-1068 phosphorylation, which was ROS-dependent and positively correlated with the extent of ROS increase and EGFRvIII downregulation. A proteomic study analyzing dynamic events of EGFR phosphorylation and ubiquitination showed the role of ROS-induced EGFR phosphorylation in shifting the outcome of EGFR from its recycling to the plasma membrane toward lysosome trafficking and downregulation [90].

Different mechanisms may affect EGFR phosphorylation such as ligand activation, internalization, recycling, and/or dephosphorylation. Auranofin-increased EGFR phosphorylation may be related to the ability of ROS to transiently inactivate protein tyrosine phosphatases (PTPs) and thereby enhance or prolong EGFR activation [91,92,93]. The inhibition of PTP activity with H_2_O_2_ was associated with increased EGFR Tyr-1068 phosphorylation [94]. H_2_O_2_ inactivates PTP1B by oxidizing its catalytic site cysteine, most likely to sulfenic acid [91]. Dagnell et al. discovered that TrxR1/NADPH directly protects PTP1B from inactivation during exposure to H_2_O_2_. The protection was blocked by auranofin and required an intact selenocysteine residue in TrxR1 [95].

ROS mediate non-canonical ligand-independent EGFR endocytosis. H_2_O_2_ at low levels activates and aberrantly phosphorylates EGFR, leading to the loss of c-Cbl-mediated ubiquitination of the receptor either directly due to Tyr-1045 hypo-phosphorylation or indirectly via Grb2. Failure of Grb2 to bind EGFR is likely due to the hypo-phosphorylation of Tyr-1068. As a result, the receptor is not targeted for clathrin-mediated internalization and degradation, thus prolonging receptor signaling and tumor progression [28,96,97].

In sharp contrast with EGFRwt, EGFRvIII is inefficiently degraded. EGFRvIII escapes downregulation due to the hypo-phosphorylation of Tyr-1045, restricted c-Cbl binding, the lack of effective ubiquitination, and reduced sorting to lysosomes [98]. EGFRvIII is defective in endocytosis despite binding to Grb2 [89], most likely due to hypo-phosphorylation of Tyr-1068. On the other hand, increased active phosphorylated EGFRvIII leads to c-Cbl ubiquitination of EGFRvIII, its downregulation and decrease oncogenic signaling [9,99]. While moderate ROS levels may be pro-tumorigenic [28,96,97], excessive ROS levels cutback EGFR-mediated cancer cell survival [23]. The potential effects of excessive ROS increase following auranofin or auranofin/L-BSO treatment on increased EGFRvIII phosphorylation, Cbl-mediated ubiquitination of EGFRvIII, its decreased stability and oncogenic properties, deserve to be explored in EGFRvIII-positive GBM.

The auranofin-induced downregulation of EGFRvIII is a striking peculiar finding, not only due to the intrinsic propensity of EGFRvIII to escape downregulation. Increasing evidence shows that the downregulation of the EGFR protein per se is the only strategy to cutback the kinase-independent pro-survival function of EGFR. For instance, EGFR downregulation decreased the pro-survival capacity of EGFR to maintain basal intracellular glucose levels and impair autophagic cell death independently from EGFR kinase activity [100]. The downregulation of the EGFR protein may also affect the pro-oncogenic roles of mitochondrial and nuclear EGFR involved in resistance to apoptosis inducers and EGFR-targeted inhibitors in GBM [101]. Nuclear EGFR promotes DNA repair [6] and acts as a transcription co-factor for STAT3/5, increasing the transcription of key genes in cell proliferation and survival [100,101]. Repurposing auranofin for the pharmacological downregulation of the EGFR protein may decrease the kinase-independent pro-survival function of EGFR along with nuclear and mitochondrial EGFR.

Auranofin-induced protein polyubiquitination was ROS-dependent irrespective of EGFR expression. Previous studies showed that auranofin inhibits proteasome-associated deubiquitinases (DUBs), which inhibits the degradation of ubiquitinated proteins and leads to the accumulation of polyubiquitinated proteins [49,50,63]. DUB proteases cleave ubiquitin from ubiquitin pro-proteins or ubiquitinated proteins. Polyubiquitination is essential for targeting misfolded and potentially detrimental proteins to the proteasome for degradation. The dysregulation of ubiquitin-specific proteases (USPs), the largest DUB family, is involved in the decreased ubiquitination of oncoproteins as a pro-survival mechanism and tumor progression of several cancers, including GBM. Of note, UBA1, a key ubiquitin-activating enzyme (E1), emerged as a novel molecular target for auranofin. Auranofin at only 100 nM directly increases the activity of UBA1. Auranofin binds to a cysteine site within the C-terminal ubiquitin domain in UBA1 and stabilizes its binding with 20 ubiquitin-conjugating enzymes (E2s). This facilitates ubiquitin charging to E2 and promotes ubiquitination by many ubiquitin ligases (E3s). These effects were exerted independently from the auranofin-induced inhibition of TrxR1 [78]. Whether auranofin inhibits DUBs or specifically increases activity of UBA1 leading to enhanced E2 and E3 ubiquitination activities in GBM is unknown. The auranofin-induced downregulation of the EGFRvIII oncoprotein and Akt may be secondary to increased polyubiquitination in EGFRvIII-positive GBM.

The Trx and GSH antioxidant pathways synergize to drive tumor progression downstream of the master redox-regulated transcription factor NRF2 [72] for over 200 antioxidant GSH and Trx genes. In response to ROS increase following chemical inhibition or genetic deletion of TrxR1, cancer cells acquire adaptive mechanisms, such as increased NRF2, to enhance the GSH antioxidant compensation mechanism and evade cell death. Increased expression of Nrf2 following auranofin treatment was ROS-dependent in the three isogenic cell lines. Auranofin at 2 μM significantly increased intracellular GSH, only in U87/EGFRwt cells. These results are in line with a proteomic study showing that auranofin increased the expression of NRF2-regulated targets including GCLC, primarily involved in GSH biosynthesis [102]. NRF2-induced compensatory GSH antioxidant activation in response to ROS increase foresees a propensity for redox resetting to decrease ROS and induce resistance to auranofin [18].

Investigating the relationship between EGFR and the GSH pathway unraveled that *EGFR* alterations in U87/EGFRwt and U87/EGFRvIII cells were associated with an intriguing decrease in baseline intracellular GSH levels compared to the U87MG cell line. Validation in TCGA/GBM patients with *EGFR* alterations showing decreased *EGFR* and *GCLC* mRNA co-expression provides new evidence for the crosstalk between EGFR alterations and the GSH pathway in GBM [31,32,33]. EGFR-positive GBM [29] and GSCs [30] exhibit high basal ROS levels, owing to increased metabolism and proliferation. Relatively low expression of GCLC in EGFR-overexpressing GBM may keep ROS at a threshold favorable for ROS tumor-promoting functions. In addition to *GCLC*, several genes involved in GSH synthesis and recycling regulate GSH antioxidant capacity in EGFR-positive GBM such as *CEBPB*, *NQO1*, and *GSTP1* [31,33]. The mechanism(s) by which EGFR may directly affect transcriptional changes in *GCLC* mRNA are unclear. The decreased transcription of GCLC and GSS through the inhibition of NRF2 emerged as a mechanism of mutant EGFR/T790M resistance to EGFR inhibition in lung cancer [103]. Conversely, EGFR silencing increased both protein and mRNA expression of GCLC in oxidative stress-induced renal injury [104]. Low antioxidant capacity in EGFR-driven GBM may confer sensitivity to combined Trx/GSH pro-oxidant strategies.

Auranofin and L-BSO synergistic activity corroborate the tenet that co-targeting Trx/GSH antioxidant systems may reach the lethal ROS threshold level in EGFR-overexpressing GBM cells. Combining auranofin with L-BSO yielded synergistic short-term and long-term cellular effects, likely due to concomitant TrxR1 inhibition and depletion of intracellular GSH in U87/EGFR cell lines. Decreased Akt phosphorylation in response to auranofin or auranofin/L-BSO combination is remarkable in the context of high basal levels of Akt activation in *PTEN*-deficient (in-frame deletion of exon 3) of U87MG cells [105]. *PTEN* loss associated with high levels of Akt activation decreases the response to anti-EGFR therapy in patients with EGFR-positive PTEN-deficient tumors [106]. *PTEN* alterations on chromosome 10 and loss of chromosome 10, commonly found in primary GBM, enhance EGFRvIII-induced signaling through hyperactivation of the PI3K/Akt pathway. Despite Akt hyperactivation in the *PTEN*-deficient U87/EGFRvIII cell line, auranofin and auranofin/L-BSO decreased EGFRvIII and Akt. The causal relationship between the overexpression of EGFRvIII and auranofin-induced downregulation of P-Akt, Akt, and EGFRvIII needs to be validated in patient-derived GBM stem cells while considering *PTEN* status.

The synergy between auranofin and L-BSO was correlated with the concomitant increase in ROS levels and histone γ-H2AX(Ser139) phosphorylation (P-γ-H2AX), a surrogate marker of DNA double-strand breaks in U87/EGFRwt and U87EGFRvIII, but not in the U87MG cell line. NAC treatment completely prevented γH2AX phosphorylation, suggesting the central role of ROS in auranofin/L-BSO synergistic oxidative DNA damage. Excessive ROS levels reach the threshold of irreversible oxidation to damage macromolecules leading to oxidative DNA damage, lipid peroxidation, and protein carbonylation. The GSH antioxidant system coordinates with DNA repair enzymes to maintain a steady-state level of ROS below the cytotoxic threshold of DNA oxidative damage. Cisplatin, a platinum-based DNA-damaging chemotherapy, inhibits the Trx system [107]. Several mechanisms account for resistance to cisplatin, including increased DNA repair activity [108], cisplatin-induced ligand-independent activation of EGFR [109], and increased intracellular GSH [110]. While resistance to cisplatin was associated with high intracellular GSH levels, glutathione depletion using L-BSO sensitized cisplatin-resistant GBM cells in vitro and in vivo [110]. Auranofin/L-BSO synergic lethality in U87/EGFRwt and U87EGFRvIII cell lines may reflect their dependence on endogenous GSH antioxidant activity to prevent DNA oxidative damage. U87MG exhibited the highest basal GSH levels and did not show P-γ-H2AX(Ser139) in response to auranofin and L-BSO combination. GSH and P-Akt decrease may be sufficient for driving auranofin/L-BSO synergistic lethality in U87MG.

While auranofin can induce oxidative DNA damage, activation of the DNA damage response may lead to drug resistance and cell survival. A sublethal dose of auranofin-induced oxidative DNA damage, followed by a strong DNA damage response activation of the DNA damage kinase Ataxia Telangiectasia and Rad3-related (ATR) in osteosarcoma and breast cancer cells. Combining auranofin and the ATR inhibitor was well tolerated and synergistically decreased tumor growth along with severe replication stress and excessive DNA breakage, reminiscent of replication catastrophe and late-stage apoptosis in vivo [111]. ROS induced DNA damage and activated the DNA damage response as a mechanism of resistance to repair the damage [112]. *EGFR* amplification has been associated with increased baseline ROS levels, and the dependence on DNA repair for cell survival, leading to increased sensitivity to a PARP inhibitor to halt double-strand break repair in patients with EGFRwt-overexpression GBM [30]. Increased ROS may contribute with decreased efficiency of DNA damage repair to the synergic lethality in U87/EGFRwt and U87EGFRvIII cell lines. Auranofin treatment led to increased Annexin V levels, suggesting that it triggers apoptosis in U87MG GBM cells [48]. Concomitant increase in ROS levels, GSH depletion, DNA damage (P-γH2AX), and PARP-1 cleavage in U87/EGFRvIII cells evokes a setting wherein excessive ROS accumulation induced DNA damage and PARP-1 activation, leading to decreased NAD+, inhibition of glycolysis, and ATP depletion [113].

Taken together, our findings align with the growing interest in testing redox-targeting strategies and developing combination therapies that exploit redox vulnerabilities in cancer [114]. Auranofin, an FDA-approved oral drug, known for its clinical safety, is able to penetrate the blood–brain barrier at doses ranging from 0.2 to 5 µmol/L [115]. Inhibition of thiol redox enzymes and inflammation pathways commonly involved in tumor growth instigated repurposing auranofin in studies, revealing the promising anticancer effects of auranofin in various cancer types [54,56,62,64,116,117,118,119,120,121] alone or as drug combination [46,47,48]. The only clinical trial using auranofin in GBM tested the combination of nine repurposed drugs including auranofin and temozolomide in the CUSP9v3 Treatment Protocol (NCT02770378) for a limited cohort of 10 patients with recurrent GBM. The most favorable outcome was observed as stable disease in six patients, while four patients experienced progressive disease [122]. Two clinical trials demonstrated the tolerability of L-BSO in neuroblastoma (NCT00002730, NCT00005835). However, thus far, L-BSO has not been approved by the FDA or the European Medicines Agency. Other studies assessed auranofin and L-BSO in mesothelioma, human lung cancer, rhabdomyosarcoma, and pancreatic cancer, yielding promising results [54,57,65,123]. Given the limited permeability of L-BSO to the central nervous system, some strategies could be adapted to increase local intracerebral administration, such as drug-impregnated wafers, Ommaya reservoir, or convection-enhanced delivery systems. The lack of effective treatments for GBM urges the need for pre-clinical testing of auranofin and L-BSO combination in orthotopic EGFR-positive GBM model in vivo to translate our findings and repurpose relatively well-tolerated drugs in future clinical trials.

## 5. Conclusions

Our study provides the first evidence for ROS-dependent sensitivity to auranofin and the synergistic interaction between auranofin and L-BSO in GBM cells harboring aberrant EGFRwt and EGFRvIII in vitro. The synergistic lethality of this combination emphasizes the significance of exploiting increased baseline redox for effective pro-oxidant strategies targeting Trx/GSH in GBM. While clinical trials failed to develop a treatment targeting EGFRvIII-positive GBM, our findings underscore the value of investigating the highly aggressive mutant EGFRvIII as a novel potential vulnerability to implement TrX/GSH co-targeting in GBM. The synergistic interaction between auranofin and L-BSO regardless of EGFR overexpression paves the way for pre-clinical in vivo studies to ultimately implement targeting the main antioxidant defense systems Trx and GSH in GBM treatment.

## Data Availability

The detailed data generated in the manuscript are openly available to the scientific community upon request.

## Supplementary Materials

The following supporting information can be downloaded at: https://www.mdpi.com/article/10.3390/cancers16132319/s1, Figure S1. Long-term effects (chronic) in colony formation assay in U87MG, U87/EGFRwt, and U87/EGFRvIII treated with auranofin (A,B) GBM cells were exposed to varying concentrations of auranofin (AU) for 72 h and then seeded for colony formation assay in the absence of the drug to assess the extended long-term cytotoxicity, over the course of 9–10 days; Figure S2. Intracellular ROS generation using auranofin in GBM EGFR variants transfected cells. (A) Relative Fluorescence Units (Fold Change) of ROS intracellular levels detected after GBM cells were treated with 3 and 6 µM AU for 2.5 h using a plate reader. (B) After treatment with 3 μM auranofin (AU) for 24 h, U87MG, U87/EGFRwt, and U87/EGFRvIII cells exhibited increased intracellular ROS levels following 30 min incubation with the general ROS indicator, CM-H2DCFDA, whereas nuclei were stained with DAPI; Figure S3. (A) N-acetylcysteine (NAC) at 1 and 2 mM for 72 h does not affect vitality of U87MG, U87/EGFRwt, and U87/EGFRvIII GBM cell lines, as shown in MTT assay. (B) Flow cytometry assay measuring viability of U87MG cells treated with 3 μM auranofin for 72 h in presence or absence of NAC; Figure S4. Fluorescence microscopy showing intracellular ROS generation using auranofin (AU) in U87MG, U87/EGFRwt, and U87/EGFRvIII treated with 3 μM AU for 24 h, with or without NAC, cells were probed with the general ROS indicator, CM-H2DCFDA. Nuclei were stained with Hoechst 33342; Figure S5. Densitometry analysis illustrates the ratio of p-EGFR (Tyr1068)/total EGFR in Figure 2G; Figure S6. Auranofin (AU) induced early and late-stage apoptosis in U87MG in a dose-dependent manner. (A) Annexin V (+), 7-AAD (−), and (B) Annexin V (+), 7-AAD (+) staining after 72 h AU (1, 3, and 5 μM treatment). Figure S7. L-buthionine-sulfoximine (L-BSO) treatment (2 and 10 µM, 72 h) does not affect vitality of U87MG, U87/EGFRwt, and U87/EGFRvIII GBM cell lines, as shown in MTT assay; Figure S8. Microscopy images showing effects on the morphology of U87MG, U87/EGFRwt, and U87/EGFRvIII cell lines for each drug combination used to analyze auranofin (AU, 1, 2, or 3 µM) and L-buthionine–sulfoximine (L-BSO, 5 µM) drug interactions; Table S1. Primary antibodies used in Western blotting analysis; Table S2. Combination index (CI) values calculated for auranofin (AU) and L-buthionine–sulfoximine (L-BSO) combination.

## Abbreviations

7-AAD	7-aminoactinomycin D
AKT	Protein kinase B (PKB)
ATP	Adenosine triphosphate
ATR	Ataxia telangiectasia and Rad3-related protein
AU	Auranofin
BCA	Bicinchoninic acid
Cbl	Casitas B-lineage Lymphoma
CI	Combination index
CM-H2DCFDA	2′,7′-dichlorodihydrofluorescein diacetate
CO_2_	Carbon dioxide
CUSP9v3	Coordinated Undermining of Survival Paths combining 9 repurposed non-oncological drugs with metronomic temozolomide-version 3
Cys	Cysteine
DAPI	4′,6-diamidino-2-phenylindole
DHE	Dihydroethidium
DMEM	Dulbecco’s Modified Eagle Medium
DMSO	Dimethyl sulfoxide
DNA	Deoxyribonucleic acid
DTNB	5,5′-dithiobis (2-nitrobenzoic) acid
DUBs	Deubiquitinases
ECL	Enhanced chemiluminescence
EGFR	Epidermal growth factor receptor
ERK	Extracellular signal-regulated kinase
FBS	Fetal bovine serum
FDA	US Food and Drug Administration
GBM	Glioblastoma
GCLC	Glutamate-cysteine ligase catalytic
Grb2	Growth Factor Receptor Bound Protein 2
GSH	Glutathione
H2AX	Histone variant H2AX
H_2_O_2_	Hydrogen Peroxide
HRP	Horseradish peroxidase
IC_50_	Half-maximal inhibitory concentration
IDH	Isocitrate dehydrogenase
kDa	Kilodaltons
L-BSO	L-buthionine-sulfoximine
MTT	3-(4,5-dimethylthiazol-2-yl)-2,5-diphenyltetrazolium bromide
NAC	N-acetylcysteine
NADPH	Nicotinamide adenine dinucleotide phosphate
NF-κB	Nuclear factor kappa-light-chain-enhancer of activated B cells
NOX3	NADPH Oxidase 3
NRF2	Nuclear factor erythroid 2–related factor 2
OXPHOS	Oxidative phosphorylation
PAGE	Polyacrylamide gel electrophoresis
PARP	Poly(ADP-ribose) polymerase
PBS	Phosphate Buffered Saline
PI3K	Phosphoinositide 3-kinase
PTEN	Phosphatase and tensin homolog
PTPs	Protein tyrosine phosphatases
PVDF	Polyvinylidene fluoride
Ras	Rat sarcoma virus (protein)
ROS	Reactive oxygen species
SDS	Sodium dodecyl sulfate
SEM	Standard error of the mean
STAT	Signal transducer and activator of transcription
TBS	Tris-Buffered Saline
TCGA	The Cancer Genome Atlas
TGX	Tris-Glycine eXtended
TMZ	Temozolomide
TNB	2-nitro-5-thiobenzoate
Trx	Thioredoxin
TrxR	Thioredoxin reductase
Tyr	Tyrosine
UBA1	Ubiquitin-like modifier activating enzyme 1
USPs	Ubiquitin-specific proteases
