# Supplementary material for: Pro-Oxidant Auranofin and Glutathione-Depleting Combination Unveils Synergistic Lethality in Glioblastoma Cells with Aberrant Epidermal Growth Factor Receptor Expression
Source: Cancers (Basel). 2024 Jun 25;16(13):2319. doi: 10.3390/cancers16132319 (PMC11240359; doi:10.3390/cancers16132319)
Supplement: Supplementary file 1 [file cancers-16-02319-s001.zip › cancers-3026189-supplementary.pdf]

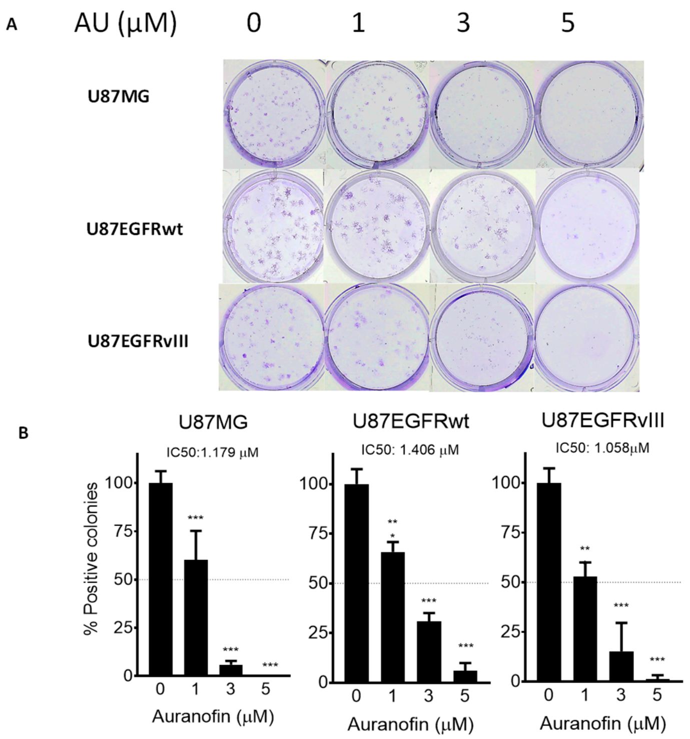

Figure S1. Long-term residual effects in colony formation assay in U87MG, U87/EGFRwt, and U87/EGFRvIII GBM cells treated with auranofin (AU) (**A-B**) Cells were exposed to varying concentrations of AU for 72 h and then seeded for colony formation assay in the absence of the drug to assess the extended residual long-term cytotoxicity, over the course of 9–10 days. Bars show the mean ± SEM (** $p < 0.01$, *** $p < 0.001$).

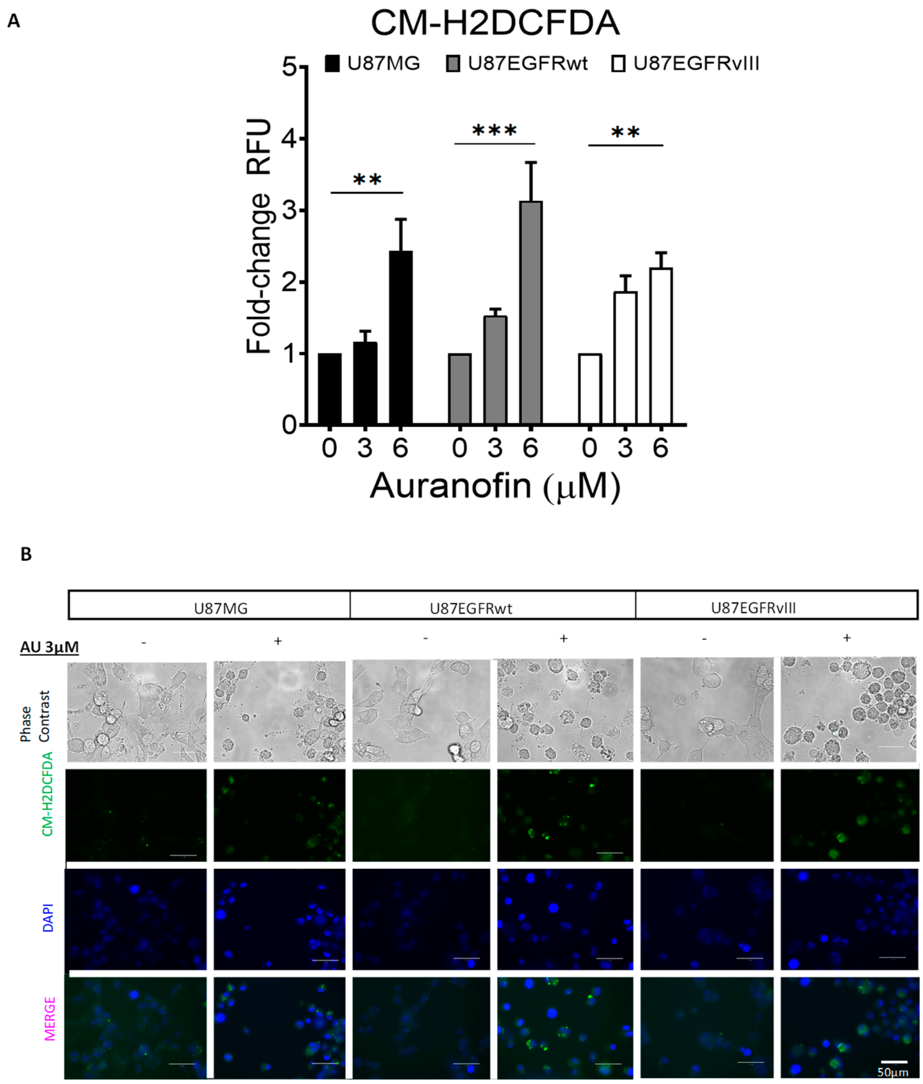

Figure S2. Intracellular ROS generation using auranofin (AU) in U87MG, U87/EGFRwt, and U87/EGFRvIII GBM cells (**A**) Relative Fluorescence Units (Fold Change) of ROS intracellular levels following treatment with 3 and 6 μM AU for 2.5 h using a microplate reader; bars show the mean ± SEM (** $p < 0.01$, *** $p < 0.001$) (**B**) AU at 3 μM for 24 h increased intracellular ROS levels detected with the general ROS indicator, CM-H2DCFDA. Nuclei were stained with DAPI.

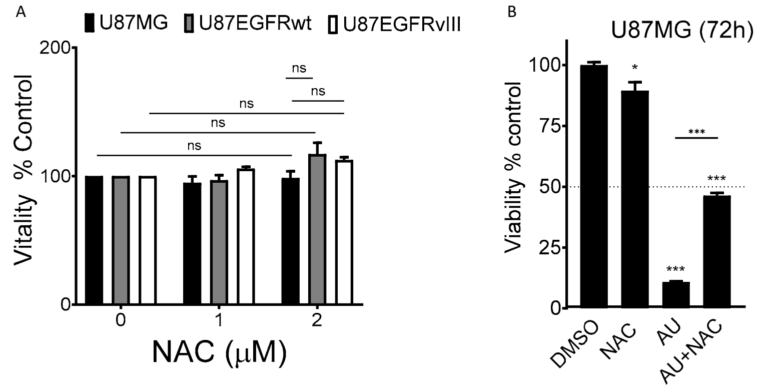

Figure S3. (**A**) N-acetylcysteine (NAC) at 1 and 2 mM for 72 h does not affect vitality of U87MG, U87/EGFRwt, and U87/EGFRvIII GBM cell lines, as shown in MTT assay; bars show the mean ± SEM ($p > 0.05$). (**B**) Flow cytometry assay

measuring viability of U87MG cells treated with 3 μM auranofin for 72 h in presence or absence of NAC; bars show the mean ± SEM (*$p < 0.05$, ***$p <$ 0.001).

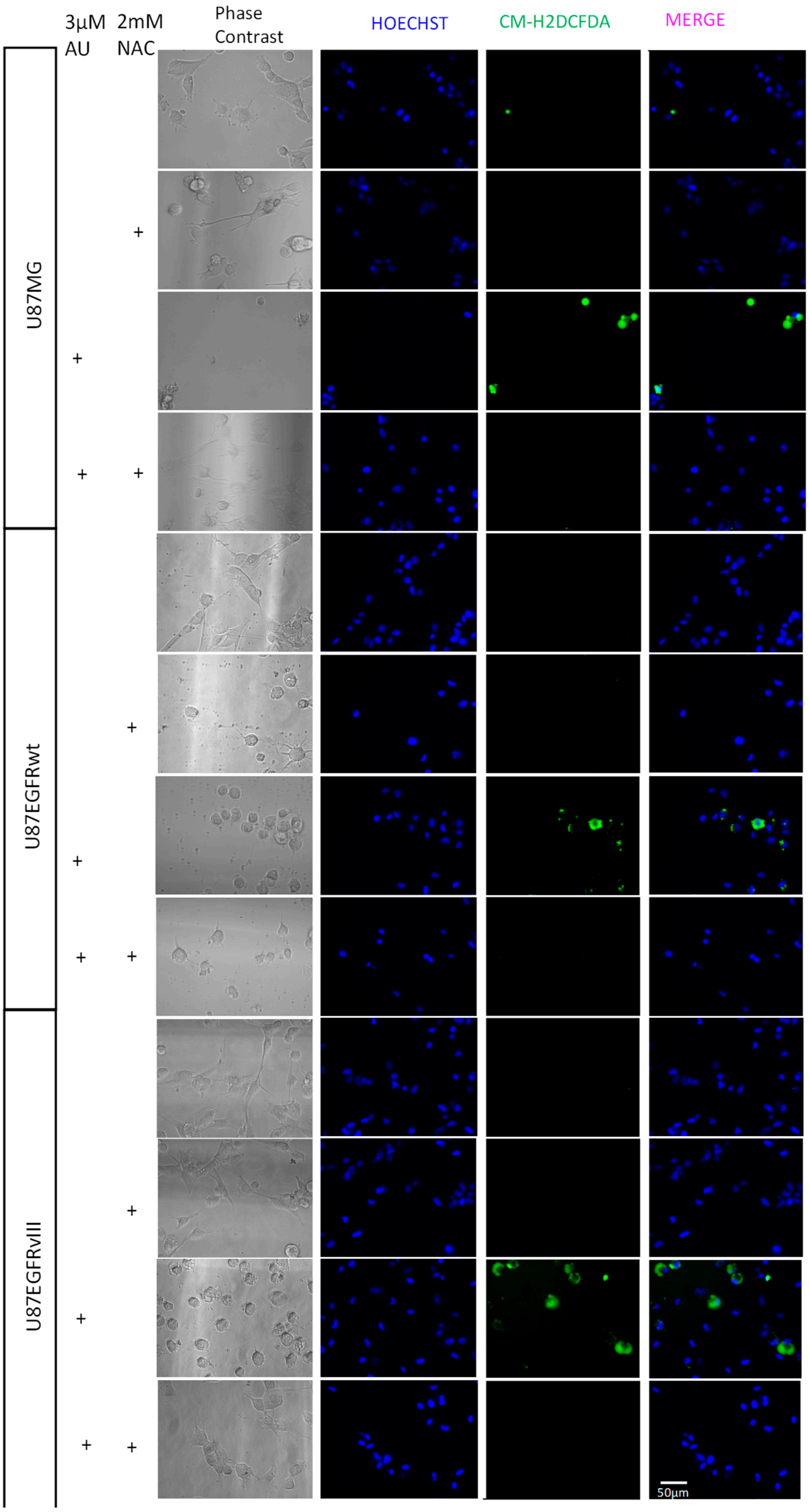

Figure S4. Fluorescence microscopy showing intracellular ROS generation using auranofin (AU) in U87MG, U87/EGFRwt, and U87/EGFRvIII treated with 3 μM AU for 24 h, with or without NAC, cells were probed with the general ROS indicator, CM-H2DCFDA. Nuclei were stained with Hoechst 33342.

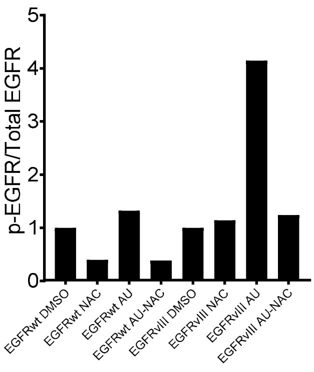

Figure S5. Densitometry analysis illustrates the ratio of p-EGFR (Tyr1068)/total EGFR in Figure 2G.

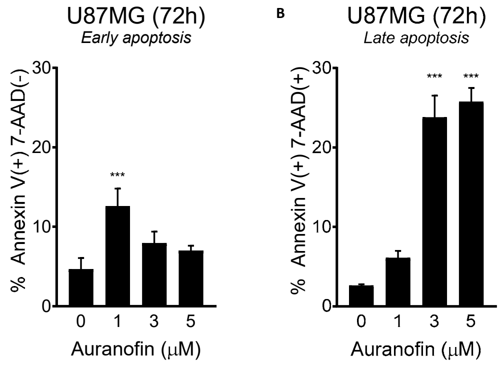

Figure S6. Auranofin (AU) induced early and late-stage apoptosis in U87MG in a dose-dependent manner. (**A**) Annexin V (+), 7-AAD (-), and (**B**) Annexin V (+), 7-AAD (+) staining after 72 h AU (1, 3, and 5 μM treatment. Bar charts show the mean ± SEM (** $p < 0.01$, *** $p < 0.001$)

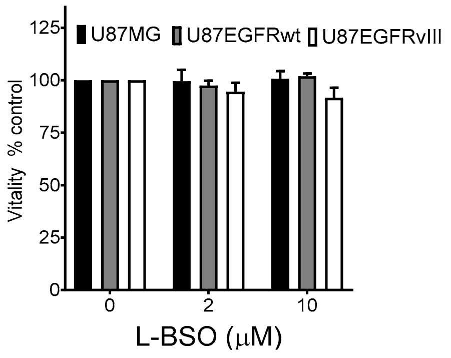

Figure S7. L-buthionine-sulfoximine (L-BSO) treatment (2 and 10 μM, 72 h) does not affect vitality of U87MG, U87/EGFRwt, and U87/EGFRvIII GBM cell lines, as shown in MTT assay.

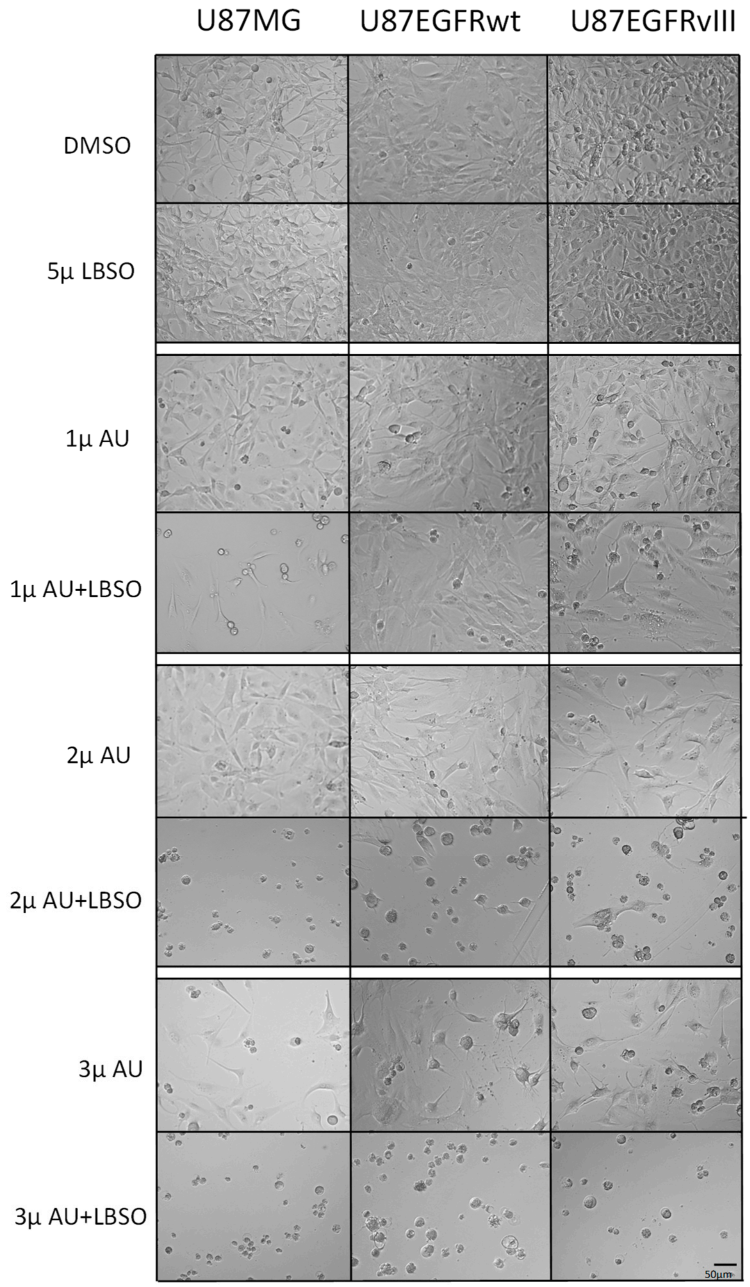

Figure S8. Microscopy images showing effects on the morphology of U87MG, U87/EGFRwt, and U87/EGFRvIII cell lines for each drug combination used to analyze auranofin (Au, 1, 2 or 3 μM) and L-buthionine-sulfoximine (L-BSO, 5 μM) drug interactions.

Table S1. Primary antibodies used in western blotting analysis.

Primary Antibody	Cat. No.	Company	Dilution
phosphorylated EGFR (p-EGFR/Y1068) (D7A5) XP®	3777S	Cell Sig-naling Technology, Danvers, MA, USA	1:1000
total EGFR (1005)	sc-03	Santa Cruz Biotechnology, Inc, Dallas, TX, USA,	1:1000
p-Akt/Ser473 (193H12)	4058S	Cell Signaling Technology	1:1000

Akt	sc-81434	Santa Cruz Biotechnology, Inc	1:500
TrxR1 (B-2)	sc-28321	Santa Cruz Biotechnology, Inc	1:1000
NRF2 (D1Z9C)	12721S	Cell Signaling Technology	1:1000
phospho-histone γH2A.X (Ser139)	05-636	EMD Millipore Corp	1:1000
ubiquitin P37	58395	Cell Signaling Technology	1:1000
β-Actin	A5441	Sigma Life science	1:10000
PARP	9542L	Cell Signaling Technology	1:1000

Table S2. Combination index (CI) values calculated for auranofin (AU) and L-buthionine-sulfoximine (L-BSO) combination.

	CI± SEM	Description
U87MG		
1μM AU + 5μM LBSO	0.540 ±0.010	Synergy
2μM AU+ 5μM LBSO	0.470 ±0.092	Synergy
3μM AU+ 5μM LBSO	0.135 ±0.093	Strong synergy
U87EGFRwt		
1μM AU + 5μM LBSO	0.441 ±0.089	Synergy
2μM AU+ 5μM LBSO	0.091 ±0.018	Strong Synergy
3μM AU+ 5μM LBSO	0.126 ±0.072	Strong Synergy
U87EGFRvIII		
1μM AU + 5μM LBSO	0.359 ±0.118	Synergy
2μM AU+ 5μM LBSO	0.302 ±0.080	Synergy
3μM AU+ 5μM LBSO	0.366 ±0.069	Synergy
